# Elucidating the Fragmentation Pathways of Cyclic and Linear Poly(dimethylpropanoate) via Tandem Mass Spectrometry and Ion Mobility Mass Spectrometry

**DOI:** 10.1021/jasms.6c00172

**Published:** 2026-10-23

**Authors:** Amman Nadeem, Calum Bochenek, Luciana R. Molina, Chrys Wesdemiotis, Scott M. Grayson

**Affiliations:** † Department of Chemistry, 5783 Tulane University, New Orleans, Louisiana 70118, United States; ‡ Department of Polymer Science and Chemistry, 1076 The University of Akron, Akron, Ohio 44325, United States

**Keywords:** Tandem mass spectrometry, IM-MS, neopentyl carbon, linear polymer, cyclic polymer, isomers, fragmentation pattern

## Abstract

More than one-half of the world’s plastic waste comes from poorly recyclable polymers, such as polyethylene and polypropylene. The increased accumulation of plastic waste is a growing environmental challenge. This led to the employment of polyesters with controlled degradation behavior for a variety of materials applications. Understanding the relationship between polymer structure and fragmentation pathways is essential for designing materials that balance durability and sustainability. Polyesters and polyamides with an C_α_ adjacent to the carbonyl, fragment via 1,5- and 1,3-hydrogen rearrangements; however, the fragmentation of a polyester where an is not present adjacent to the carbonyl has not been researched thoroughly. This work reports the first detailed characterization of the fragmentation behavior of both linear and cyclic poly­(dimethylpropanoate) (poly­(DMP)). Poly­(DMP) possesses a neopentyl carbon which slows degradation and inhibits the typical 1,5-hydrogen rearrangements common in other polyesters. Cyclic poly­(DMP) undergoes ring opening at various points before fragment generation, resulting in a mixture of ring-opened linear species, while the linear polymer predominantly fragments through single-bond cleavages. Additionally, the fragmentation patterns are influenced by poly­(DMP) end groups, with distinct patterns observed for hydroxyl-terminated, halogen-terminated, and pyridinium-initiated polymers. Moreover, ion mobility mass spectrometry (IM-MS) effectively distinguishes isomeric fragments in cyclic poly­(DMP) based on the differences in the collision cross-section of cyclic precursor and the resulting linear fragment ions.

## Graphical abstract

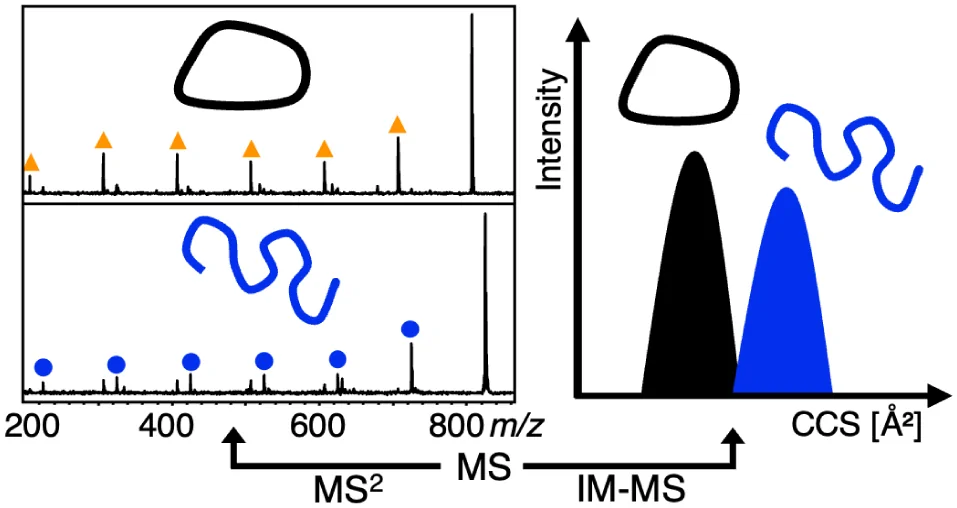

## Introduction

Synthetic polymers have become essential materials in modern society with widespread use in packaging, textiles, medical devices, and countless other applications. Their extensive use has provided significant advancements in modern society due to their durability, versatility, and affordability.^1^ Nevertheless, their environmental longevity has become a growing concern. Commodity polymers such as polyethylene and polypropylene contain chemically stable carbon–carbon backbones that provide excellent durability. However, these polyolefins are highly resistant to degradation, accumulating in landfills, and contributing significantly to the global plastic pollution crisis.^2–4^ In contrast, biodegradable polyesters such as polylactide (PLA), polyhydroxyalkanoates, and polycaprolactone (PCL) contain ester linkages that hydrolyze and degrade under appropriate conditions, making them attractive alternatives for more sustainable materials.^5,6^ The degradability of polyesters is influenced by the accessibility of their ester linkages to water and other degrading agents. PLA and PCL possess relatively unhindered ester bonds that are amenable to chain scission, but incorporating sterically bulky substituents close to the ester linkages can dramatically reduce the bond cleavage while maintaining the presence of chemically degradable functional groups.^7^

These polyester materials can be tailored to exhibit specific properties such as strength, elasticity, or biodegradability depending on factors including molecular weight, structure, topology, chemical composition, and functionalization.^8^ To understand and evaluate these polyesters, a variety of characterization techniques are commonly used, including nuclear magnetic resonance (NMR), size exclusion chromatography (SEC), viscometry, differential scanning calorimetry (DSC), and thermogravimetric analysis (TGA).^9–11^ However, while these methods are effective in measuring physical and chemical properties, they often provide limited information about the isomeric architectures and end groups of the polymers. For instance, as polymer chains grow longer and the degree of polymerization increases, the signals from end groups in NMR spectra become much less prominent.

Mass spectrometry (MS) serves as a valuable complementary tool in polymer analysis. It works by converting analyte molecules into gas-phase ions and separating them based on their mass-to-charge ratio (*m*/*z*), offering detailed insights into the molecular structure that other techniques may not reveal.^12^ Matrix-assisted laser desorption/ionization (MALDI) and electrospray ionization (ESI) MS are soft ionization techniques, meaning they do not cause extensive fragmentation for most polymers. This is important because the loss of end groups or repeat units through fragmentation can result in the loss of valuable chemical information.^13^ Matrix-assisted laser desorption/ionization time-of-flight (MALDI-ToF) MS is a powerful and efficient method for analyzing a wide range of polymers, and it typically requires mixing a specific ratio of matrix, polymer, and salt for sample preparation to obtain precise results. It offers high resolution, as opposed to SEC, allowing for the clear observation of isotopic patterns in polymer peaks and precise measurement of the analyte’s mass, if the dispersity (*Đ*) is below 1.2.

Polymerizations can lead to the formation of various side products (impurities), depending on the reaction conditions. MALDI-ToF MS has proven to be remarkable in not only distinguishing between the impurities but also providing information on the repeat units, total end groups, and molecular weight distributions.^14,15^ Unfortunately, MS alone fails to distinguish between different polymeric architectures. Tandem mass spectrometry (MS^2^), on the other hand, can provide information on individual end groups,^16–19^ distinguishing isomeric copolymers (meta vs para side chains) through distinct fragmentation patterns,^20^ decipher copolymer sequences,^21–24^ and reveal macromolecular architectures and topologies.^14,20,25–27^ For example, it is difficult to differentiate between poly­(*n*-butyl acrylate) and poly­(*t*-butyl acrylate). Since both polymers have identical molecular weights, they appear as identical peaks in the MS spectrum. However, the distinct fragmentation patterns in MS^2^ allow them to be differentiated as their respective isomeric architectures.^28^

Cyclic polymers have shown to possess unique properties compared to their linear analogs. Because of the absence of chain ends, cyclic polymers show significantly different material characteristics, such as higher glass transition temperatures (*T*_g_) and melt transition temperatures (*T*_m_), reduced hydrodynamic volumes, increased crystallization rates and thermally stable crystalline domains, lower intrinsic viscosities, and higher refractive indexes.^29–32^ It is always useful to shed light on the structural differences between cyclic and linear polymers. There are many examples of MS^2^ on linear polymers, but MS^2^ of cyclic polymers is gaining interest because it requires two homolytic cleavages to form a fragment. Osaka and coworkers used tandem mass spectrometry to compare the fragmentation patterns of cyclic and linear poly­(lactic acid) and to examine the effect of solvolysis on the fragmentation of these two species. Linear poly­(lactide) fragmented into three different products, whereas cyclic produced a single dominant fragmentation product.^33^ Rizzarelli and Puglisi reported a linear and cyclic poly­(ester amide) synthesized via melt condensation of sebacic acid and 4-amino-1-butanol. Tandem mass spectrometry provided structural insights into the ester/amide sequences along the polymer chains. The dominant product ions indicated that fragmentation primarily occurs via a β-hydrogen transfer rearrangement, resulting in selective cleavage of the −O–CH_2_– bonds.^34^

Moreover, Alexander and others made a macrocyclic polyester copolymer and employed tandem mass spectrometry to deduce the fragmentation patterns of cyclic and linear polymers. Macrocyclic copolyesters fragmented first by ring opening at the ester groups through a 1,5-hydrogen rearrangement while preserving sequence information that allowed analysis of comonomer order. Linear analogues showed similar sequence behavior even without a ring opening step, and both systems mainly displayed block architectures because of different reactivities of the comonomers. MS^2^ therefore provided valuable information connecting copolymer sequence and structure to their thermoresponsive properties.^35^ In addition to polyesters and polyamides, MS^2^ has also been applied to a variety of polymers to differentiate between cyclic and linear architectures based on their unique fragmentation patterns.^36–38^

Most polyesters and polyamides contain an C_α_ adjacent to ester or amide functional groups, so these polymers commonly undergo fragmentation through 1,5- and 1,3-hydrogen transfer pathways. A 1,5-rearrangement in polyesters or polyamides is a gas-phase fragmentation mechanism involving intramolecular transfer of a γ-hydrogen (relative to the carbonyl of the ester) through a six-membered transition state, leading to ester bond cleavage and formation of characteristic fragment ions. It has been evident from the literature that the majority of the polyesters or polyamides typically undergo facile 1,5-proton rearrangements;^39–41^ however, this is not possible with poly­(dimethylpropanoate) (poly­(DMP)) as it has a neopentyl carbon that lacks the substantial protons on C_5_ for 1,5-proton rearrangement. The neopentyl carbon also increases steric hindrance around the functional group and reduces the accessibility of the ester bond to hydrolytic attack, potentially decreasing its susceptibility to degradation.

For the first time, MS^2^ was used to investigate the fragmentation patterns of poly­(DMP). The polymer backbone was kept constant, while only the end groups were varied to study their influence on fragmentation, including hydroxyl, bromo, chloro, and pyridinium functional groups, or removal of the entire end group in the case of cyclic polymers. It was observed that various fragmentation products emerged depending on the end group atom size (OH < Cl < Br) in the linear polymer or lack thereof in the cyclic polymer. IM-MS was further employed to distinguish the isomeric species observed in the MS^2^ spectrum of cyclic poly­(DMP).

## Results and Discussion

A mixture of low molecular weight cyclic and linear poly­(DMP) was formed by reacting 2,2-dimethyl-3-hydroxypropionic acid (HPA) in the presence of a catalytic amount of *para*-toluenesulfonic acid (*p*-TsOH). The polymers were formed via condensation polymerization. These experiments were done at temperatures above 100 °C to remove the water vapors formed so that the reaction is continuous until workup (Scheme SI 1). Linear products possess a carboxylic acid and a hydroxyl on the end groups. Cyclic polymers were formed by the loss of a water molecule to ring-close the polymer. As the temperature increased, the cyclic species increased in the sample. This happened because the chain end group attacks either the initiator or some other ester on the same chain to close the ring. The absence of methylene protons on ^1^H NMR and a peak with a loss of 18 Da (water) on MALDI-ToF MS confirmed the presence of cyclic poly­(DMP). Both these polymers were characterized using ^1^H NMR, FTIR, and MALDI-ToF MS.

In MALDI-ToF MS, carboxylic acid end groups are commonly detected as metal-adducted species due to deprotonation and coordination of alkali metal cations.^39,42^ This can be seen in Figure SI 1 where the proton in the COOH group gets replaced by Li^+^ forming COO^–^Li^+^; however, the product requires an extra Li^+^ ion to ionize and to reach the detector. Moreover, MS^2^ is assisted by the Lewis acidic nature of the Li^+^ ion. Coordination of Li^+^ to the oxygen atoms of C=O bonds forms a well-stabilized charge site, thereby facilitating charge-remote homolytic cleavages. Li^+^ cation was chosen for this study because the spectrum had better signal-to-noise ratio especially for the low-intensity fragments in the baseline.

### MS^2^ of Linear Poly­(DMP)–OH

The MALDI-ToF MS spectrum of the linear polymer is shown in Figure SI 1. Two product distributions were observed in the spectrum. The lower-intensity distribution (A_n_) corresponds to species in which the proton of the terminal carboxylic acid (COOH) group is intact, and it ionizes with Li^+^ as the cation, whereas the higher-intensity distribution (B_n_) has this proton removed, and it is replaced as the lithium carboxylate, plus ionizes with a second lithium as the cation. The 10-mer precursor of the linear poly­(DMP) was fragmented by MALDI-ToF MS^2^ into five different products (Figure 1). The major fragment distribution, denoted by blue circles, resulted from the loss of several repeat units from the precursor ions. However, in this study, the same product can be formed via three different bond cleavages. Specifically, the major distribution can arise by CH_2_—OC=O cleavage followed by a 1,2-methyl shift, an O—C=O ester bond breakage, and a OC=O—C­(CH_3_)_2_ bond breakage (Scheme 1A, 1B, and 1C, respectively).

**Figure 1:**
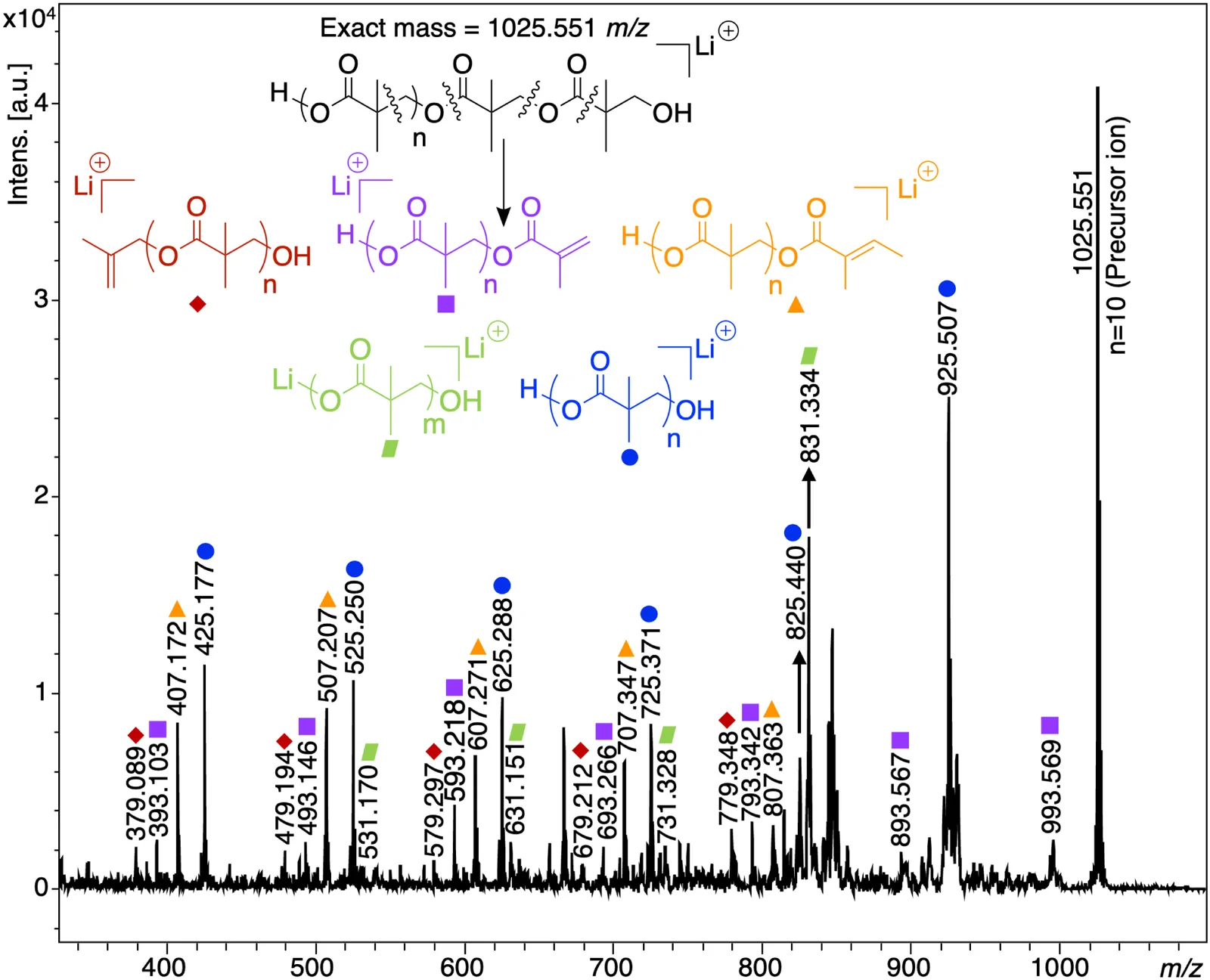
MALDI-ToF MS^2^ mass spectra of the lithiated linear poly­(DMP)–OH 10-mer precursor and its five different fragmentation products. The fragments marked with green rhombus originate from coadmitted (not resolved) dilithiated 10-mer (see text).

**Scheme 1:**
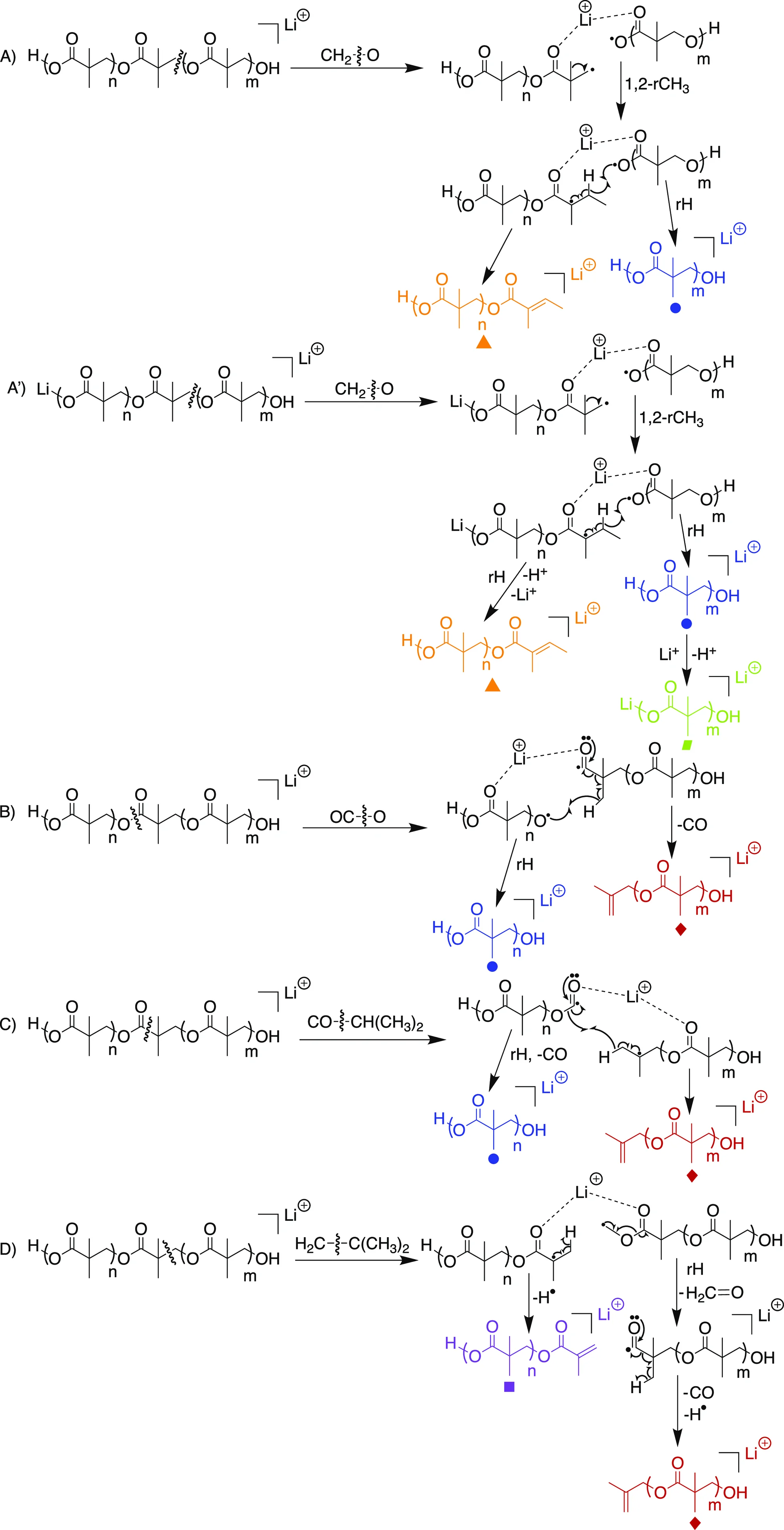
Fragmentation Mechanism of the Linear Poly­(DMP)–OH, where *n* and *m* Denote the Number of Repeat Units^a^

a Radical-induced fragmentation hydrogen pathway leading to products, where 1,2-rCH_3_and rH designate a 1,2-methyl shift and a hydrogen transfer, respectively. Different bond cleavages result in the formation of distinct fragment ions. A) Cleavage of the methylene C–O bond in the precursor poly­(DMP)–OH ion, A′) cleavage of the methylene C–O bond in the poly­(DMP)–OH ion contaminated by Li^+^, B) cleavage of the ester linkage, C) cleavage of the bond between the carbonyl carbon and the quaternary carbon, and D) cleavage of the bond between the methylene carbon and the quaternary carbon. The orange alkene product (triangles) can be produced as *E* or *Z* isomer; however, the one shown has a lower heat of hydrogenation and is more favorable.

Cleavage of CH_2_—OC=O followed by a 1,2-methyl shift^43,44^ produces two different products, a water-terminated (blue circles) and an alkene-terminated (orange triangles) poly­(DMP) (Scheme 1A). This bond cleavage initially generates a primary radical, which is subsequently converted into a more stable tertiary radical through a 1,2-methyl shift. A further homolytic cleavage of the C–H bond generates a C=C bond on one fragment, while simultaneously converting the acyloxyl radical, via proton abstraction, to a carboxyl group in the other resulting fragment. The orange alkene product (triangles) can be produced as *E* or *Z* isomer, however, the one shown has a lower heat of hydrogenation and is more favorable. This fragmentation process is confirmed by similar work of Gies et al. as they investigated the fragmentation pathways of poly­(butylene adipate), demonstrating that a 1,5-hydrogen transfer and a homolytic cleavage generates two fragments: one containing a C=C bond and the other bearing a carboxylic acid group.^45^ Interestingly, the distribution labeled by green rhombus is shown in Scheme 1A′. It forms through the same mechanism as in Scheme 1A. This is attributed to contamination of the selected lithiated poly­(DMP)–OH 10-mer with dilithiated 10-mer which is more abundant (Figure SI 1). These two precursor ions differ in mass by only 6 Da and cannot be well resolved under MALDI-ToF MS^2^ conditions, where a timed ion selector (TIS) is used for precursor ion isolation (vide supra).

Alexander et al. previously demonstrated that the ester bond scission is the primary fragmentation mechanism in both linear and cyclic polyester copolymers which proceeds by a 1,5-proton transfer. The fragments formed possessed a C=C and a COOH at the end groups.^35^ Esters are labile functional groups, and cleavage of the O—C=O single bond eventually generates a hydroxyl-terminated poly­(DMP) fragment and a disubstituted alkene, represented by red diamonds. Ester bond cleavage results in the formation of an alkoxy radical intermediate and an acyl radical intermediate (Scheme 1B). The alkoxy radical can abstract a hydrogen atom from the fragment containing an acyl radical to form an OH-terminated fragment, whereas the elimination of carbon monoxide from the acyl radical after the loss of a hydrogen radical yields a C=C bond. Loss of carbon monoxide is a known phenomenon in polyamide and polyester fragmentation.^46,47^

Furthermore, the breaking of OC=O—C­(CH_3_)_2_ bond in Scheme 1C yields the same products, a hydroxyl-terminated fragment and a disubstituted alkene, as shown in Scheme 1B; however, the mechanism adopted here is slightly different. In this case, bond cleavage results in the formation of an alkoxycarbonyl radical intermediate and an alkyl radical intermediate. The alkoxycarbonyl radical abstracts a hydrogen atom and loses carbon monoxide to form an OH-terminated fragment, whereas loss of a H^•^ atom from the alkyl radical yields a C=C bond. Both mechanisms in Scheme 1B and 1C are plausible and can occur concurrently.

Homolytic cleavage of a carbon–carbon bond is a known phenomenon,^48,49^ but not when the bond is adjacent to a quaternary carbon center. In Scheme 1D, a C–C scission generates a tertiary radical and a methyl ester radical intermediate. The methyl ester radical can subsequently eliminate formaldehyde, carbon monoxide, and a hydrogen atom to yield the disubstituted alkene product (red diamonds). The purple product distribution (squares) results from hydrogen radical loss from the tertiary radical, forming a methacrylate.

Generally, the C–O bond is stronger compared to the C–C bond; however, its cleavage might be kinetically favorable; hence, the blue product is dominant in the spectrum.

### MS^2^ of Cyclic Poly­(DMP)

The next polymer characterized via MS^2^ is the cyclic polymer. In an MS^2^ experiment, a cyclic polymer needs to undergo a ring opening and a scission before it can generate smaller fragments that reveal structural information. This ring opening can happen at various points along the polymer chain.^35^ The MALDI-ToF MS spectrum of the cyclic poly­(DMP) is shown in Figure SI 2. Two product distributions are observed in the spectrum. The higher-intensity distribution, denoted by A_n_, corresponds to cyclic polymers ionizing with Li^+^ ions, while the lower-intensity distribution, labeled with pink A_n′_, represents cyclic polymers ionizing with Na^+^ ions. The sample also contains a high-molecular-weight linear product with OH and COOH end groups. However, MALDI-ToF MS is known to exhibit a molecular weight bias when analyzing broadly dispersed samples.^50,51^ As a result, the high-molecular-weight linear species are difficult to observe in the spectrum. The octamer cyclic DMP (*n* = 8) was chosen as the precursor ion to fragment via MS^2^.

The octamer fragments into three distinct products highlighted by red diamonds, orange triangles, and blue circles in Figure 2. These species arise from an initial bond cleavage that results in linear products. The linear products further break down into smaller linear species. Two possible bond cleavages are observed, either at the C—O=O ester linkage or at the CH_2_—OC=O linkage, resulting in two distinct linear intermediates.

**Figure 2:**
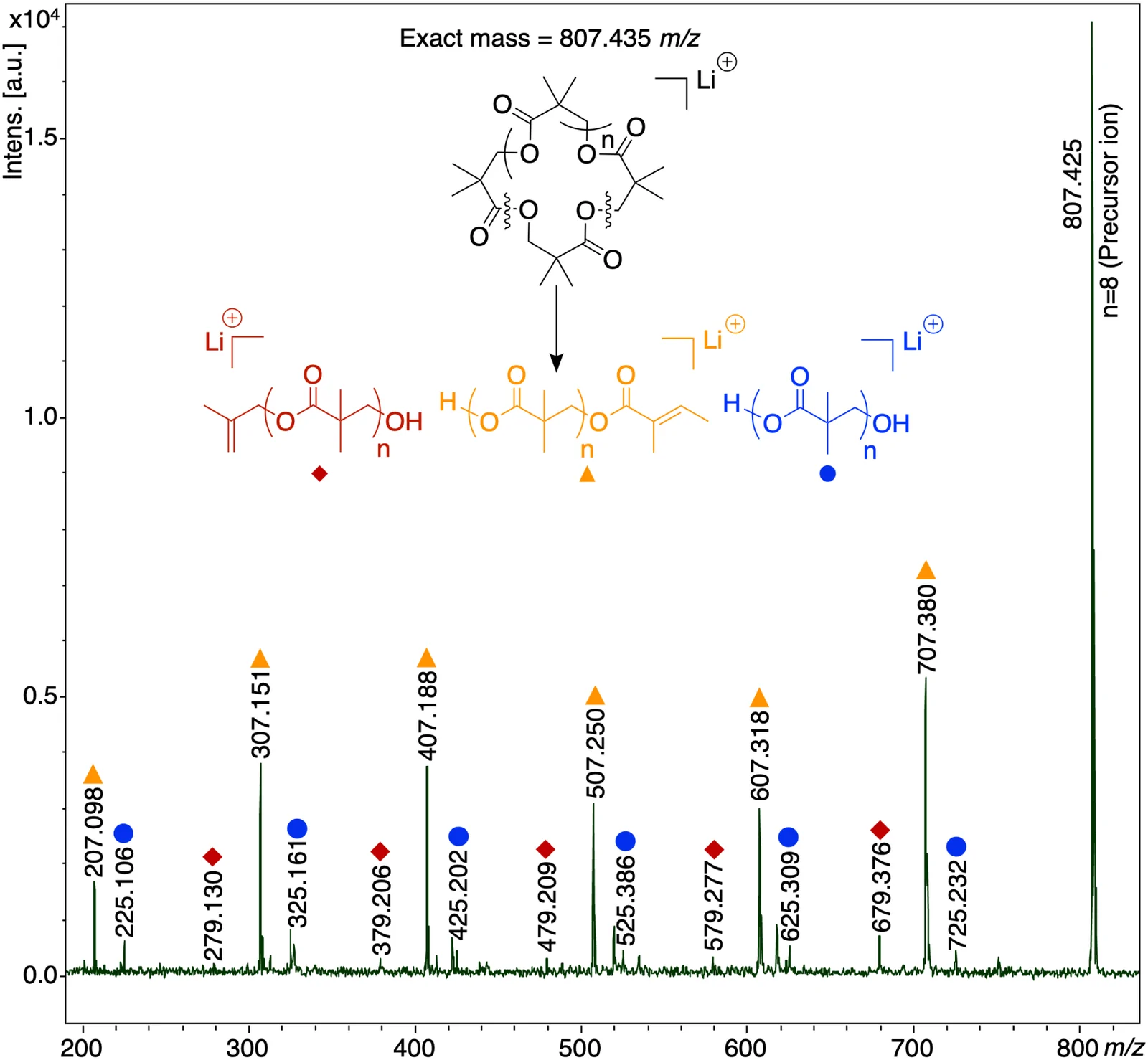
MALDI-ToF MS^2^ mass spectrum of the lithiated cyclic poly­(DMP) 8-mer precursor and its three different fragmentation products.

First, CH_2_—OC=O cleavage^43^ generates a linear product with a trisubstituted alkene at one end group (Scheme 2A). This product, represented by orange triangles, is the major distribution for the cyclic material, in contrast to the linear poly­(DMP)–OH (Figure 1), where the water-terminated product (blue circles) was dominant. The higher abundance of the trisubstituted alkene product arises because it is formed through a consecutive fragmentation via the same mechanism that yields two smaller products with identical chemical structures. The orange alkene product (triangles) can be produced as an *E* or *Z* isomer; however, the one shown has a lower heat of hydrogenation and is more favorable. The orange ring-opened intermediate can also undergo an alternative cleavage involving the loss of CO^47^ to form the water-terminated linear product (blue circles). The mechanism is shown in Scheme SI 2A.

**Scheme 2:**
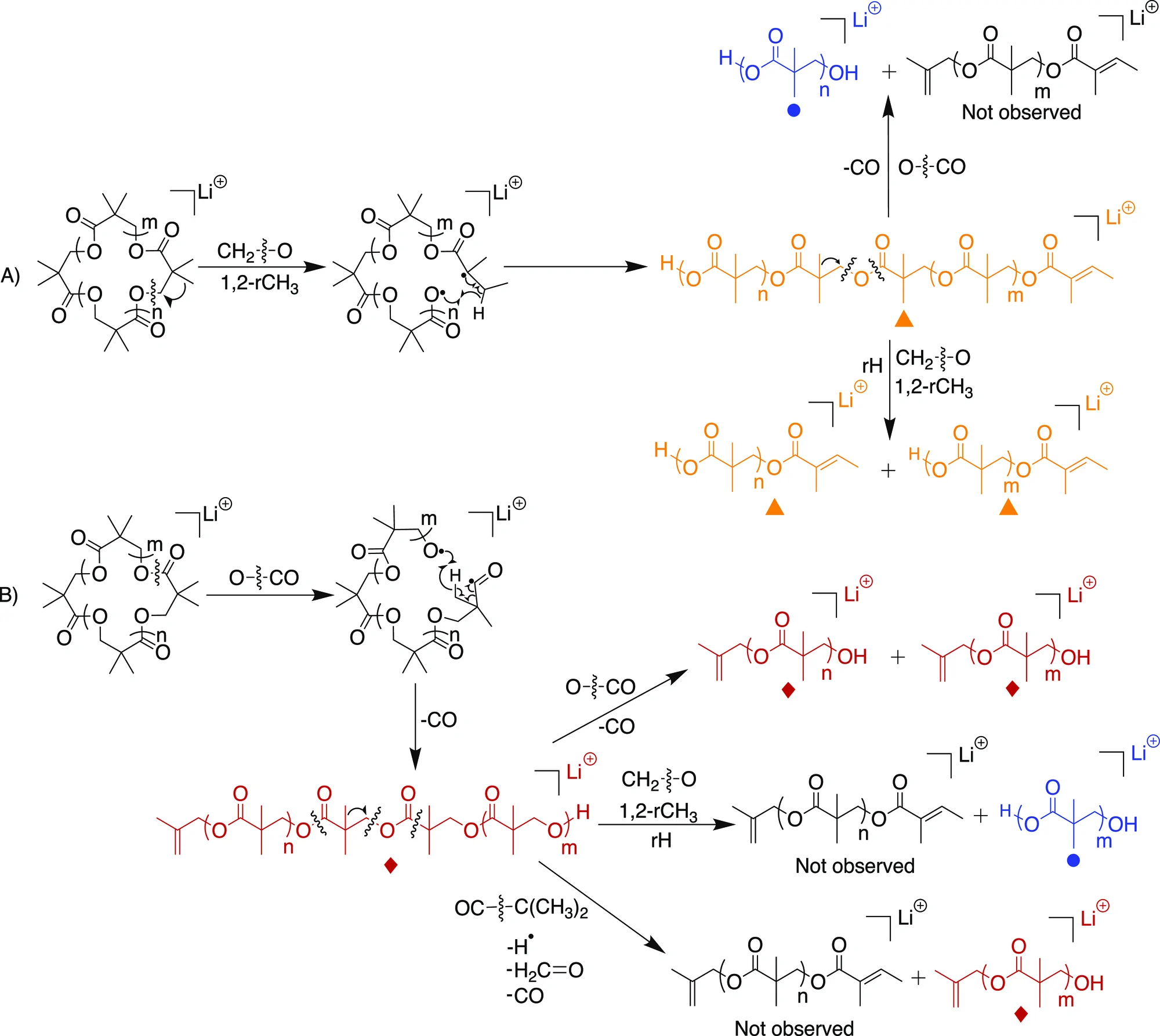
Fragmentation Scheme of Cyclic Poly­(DMP), where *n* and *m* Denote the Number of Repeat Units^a^

a Radical-induced fragment dissociation pathways leading to products, where 1,2-rCH_3_and rH designate a 1,2-methyl shift and a hydrogen atom transfer, respectively. There are two bond cleavage pathways: A) cleavage of the methylene C–O bond and B) cleavage of the ester linkage. The orange alkene product (triangles) can be produced as *E* or *Z* isomer; however, the one shown is more favorable.

The second linear intermediate formed is the red product, denoted by diamonds, which results from cleavage at the ester linkage. This intermediate can then undergo O—C=O ester, CH_2_—OC=O, and OC=O—C­(CH_3_)_2_ bond cleavages, generating either smaller fragments of identical chemical structure (red diamonds) or species bearing alkene end groups at both termini (black). The latter product was not detected in the spectra, likely due to its nonpolar end groups and hence lower Li^+^ affinity.

There are substantial differences in the cation affinities of the product ions formed during MS^2^. Rizzarelli and Puglisi demonstrated that upon fragmentation, the cation can remain on either of the two fragments; however, the relative cation affinities of the fragments govern how the cation is distributed among the resulting species, thereby determining which fragments are detected.^34^ Fragment ion formation is further influenced by the mass-to-charge ratio of the precursor ion, the nature of the cation, and the mobility of the polymer chain.^52^ Moreover, Puglisi and coworkers showed that polymer end groups play a dominant role in MALDI-ToF ionization efficiency and strongly affect the relative intensities of detected oligomer peaks, particularly for polymers or blends with similar molecular weights and dispersities. They proved that COOH end groups have better affinity for cations and subsequently higher intensities compared to OH end groups.^53^

Ion mobility mass spectrometry separates ions based on their size, shape, and charge by measuring their drift through an inert gas under an electric field, allowing calculation of their collision cross-section (CCS) area.^54^ Ion mobility decreases as the collision cross-section increases; therefore, the experimentally measured mobility can be used to infer detailed information about an ion’s size and structural characteristics.^55^ Coupled with mass spectrometry, this technique provides detailed insights into the structural conformations, composition, and dynamics of ions, such as in host–guest interactions,^56,57^ proteins,^58^ synthetic polymers,^59,60^ and supramolecular assemblies and clusters.^61,62^

MALDI-ToF MS^2^ was used to obtain the spectrum shown in Figure 2. The major fragment distribution, marked with orange triangles, all arise from the same precursor ion (Scheme 2A). ESI-MS^2^ was then used to obtain the spectrum in Figure 3A using Na^+^ instead of Li^+^. ESI predominantly produces sodiated ions due to trace sodium present in solvents and glassware, even when lithium salt is added. Therefore, Na^+^ was used in the ESI experiments to minimize the spectral complexity and isobaric overlaps. Previous studies have shown that lithiated and sodiated precursor ions exhibit nearly identical MS^2^ fragmentation behavior under both MALDI and ESI conditions.^43^

**Figure 3:**
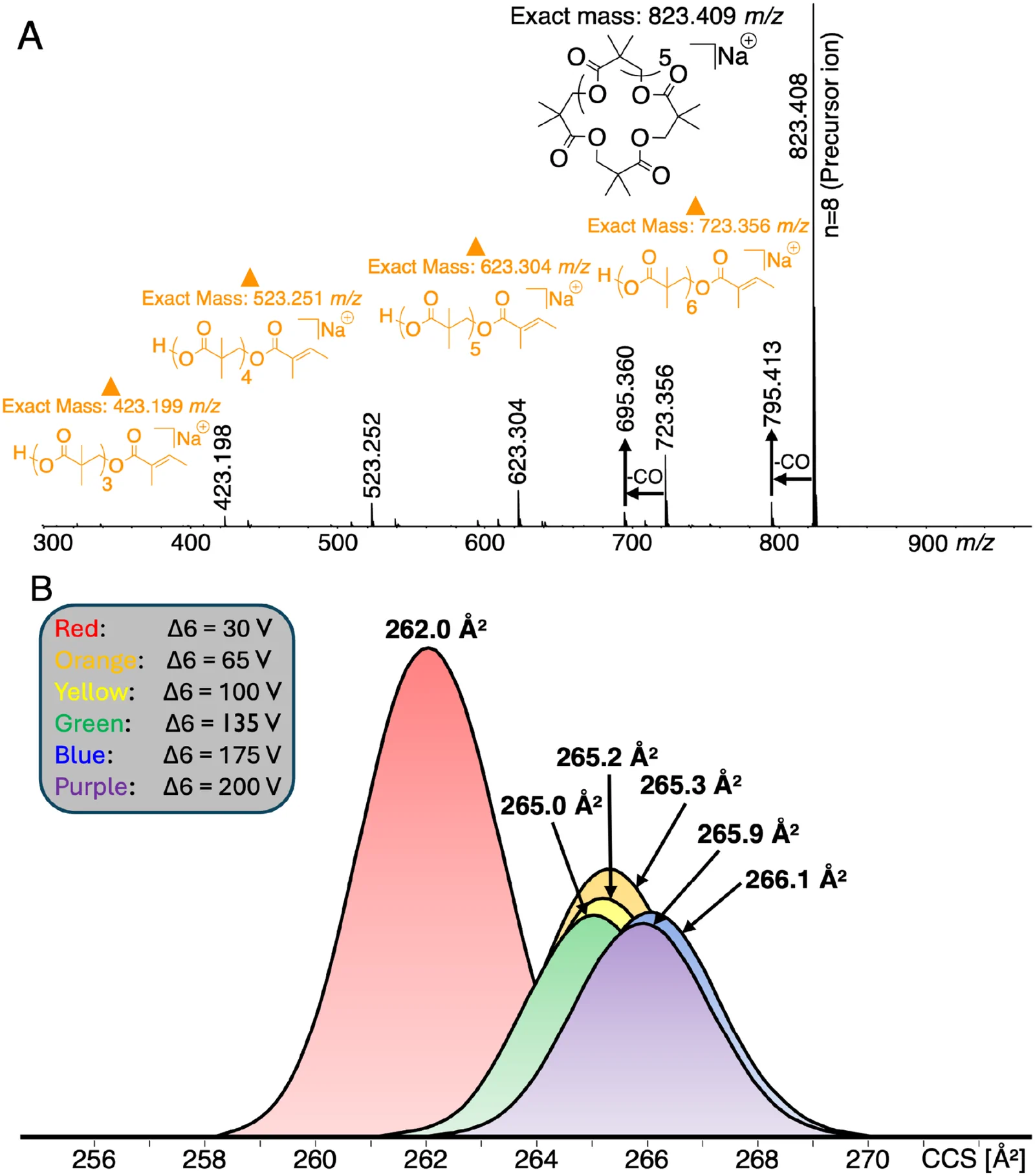
(A) Electrospray ionization MS^2^ (ESI-MS^2^) spectrum of the 8-mer cyclic poly­(DMP) with sodium cation and (B) ion mobility mass spectrometry (IM-MS) spectrum of this 8-mer. The CCS increases as the voltage used to collisionally activate the cyclic 8-mer increases indicating the occurrence of bond cleavage and the ring opening of macrocyclic into a linear species, the orange alkene fragments (triangles) shown in Scheme 2A. This product can be produced as *E* or *Z* isomer; however, the one shown has a lower heat of hydrogenation and is more favorable.

ESI-MS^2^ also showed a similar trend to MALDI-MS^2^. This raised the question of whether the observed fragments are produced directly from the cyclic structure without an alkene chain end or from linear chains containing an alkene. To address this, IM-MS was applied. Figure 3B shows the spectrum of the cyclic poly­(DMP) precursor ion (*n* = 8) after collisional activation under different applied voltages. The red peak at CCS = 262 Å^2^ corresponds to the initial intact cyclic precursor. As the voltage increased above 65 V, this peak shifted to higher CCS values (265–266 Å^2^). The increase in CCS indicates that the ring opened, producing a linear chain.^63^ Because linear chains are longer and less compact than cyclic molecules, their peaks appear at higher CCS values in IM-MS spectra. The relatively small increase in CCS suggests that the linear product remains partially compact in the gas phase due to intramolecular folding, stabilized by coordination of the Na^+^ ion with multiple carbonyl groups. In contrast, Figure SI 3 shows the ESI-MS^2^ and IM-MS of an 8-mer linear poly­(DMP) precursor ion after collisional activation under different applied voltages. The red peak at CCS = 270 Å^2^ corresponds to the initial linear precursor ion (841.417 *m*/*z*). As the voltage increased, the fragmented peaks remained essentially at the same position as the initial precursor ion, with no significant shift in CCS as seen with the cyclic precursor ion. This is because the linear polymer architecture remains intact in its less compact conformation. The CCS values for the linear ion chains produced from the cyclic precursor (Figure 3B) are between 265 and 266 Å^2^, whereas the CCS values for the linear ion fragments produced from the linear precursor (Figure SI 3B) are between 269 and 270 Å^2^. This difference in CCS values is attributed to the difference in the exact mass of the precursor ions. The cyclic precursor lacks end groups and has an *m*/*z* of 823.408, whereas the linear precursor contains end groups (water = 18 *m*/*z*) and has an *m*/*z* of 841.717. These results confirm the formation of linear poly­(DMP) fragments following MS^2^ of cyclic precursors. The same trend was observed in cyclic precursors with *n* = 7 and 9 (Figure SI 4 and Figure SI 5).

The major fragment distributions observed in Figures 2 and 3A originate from orange linear intermediate (triangles) shown in Scheme 2A. Because the cyclic polymer lacks chain-end functionalities and contains a neopentyl carbon, the dominant MS^2^ fragmentation pathway involves cleavage of the CH_2_—OC=O bond, followed by a 1,2-methyl shift (Scheme 2A). Notably, cleavage of the CH_2_—OC=O linkage is favored over scission of the O—C=O ester bond, as seen by the predominance of the orange triangle fragment ions relative to the red diamond or blue circle fragment ions in the spectrum of Figure 2.

### MS^2^ of Poly­(DMP)-Cl

Next, a linear poly­(DMP) bearing a chlorine atom in place of the terminal hydroxyl group (poly­(DMP)-Cl) is studied. One equivalent of monomer, 3-chloro-2,2-dimethylpropanoic acid, was reacted with two equivalents of potassium carbonate in the presence of dimethylformamide to produce poly­(DMP)-Cl. The product was characterized using ^1^H NMR and MALDI-ToF MS.

The MALDI-ToF mass spectrum of the precursor linear poly­(DMP)-Cl is shown in Figure SI 6. Two distinct product distributions were observed. One distribution, denoted by A_n_, corresponds to species in which the proton of the terminal carboxylic acid (COOH) group is intact, and it ionizes with Li^+^ as the cation, whereas the second distribution, marked with B_n_, has the COOH group replaced by the corresponding lithium carboxylate, and it ionizes with a second lithium as the cation.

Poly­(DMP)-Cl was fragmented via MS^2^ into six different products, as shown for the lithiated 8-mer (*m*/*z* 843.445 Da) in Figure 4. The mechanism leading to these products is shown in Scheme SI 3. The characteristic isotopic pattern of one chlorine, arising from the natural abundances of ^35^Cl (100%) and ^37^Cl (32%), is manifested by the observation of M^+^ and [M+2]^+^ peaks.

**Figure 4:**
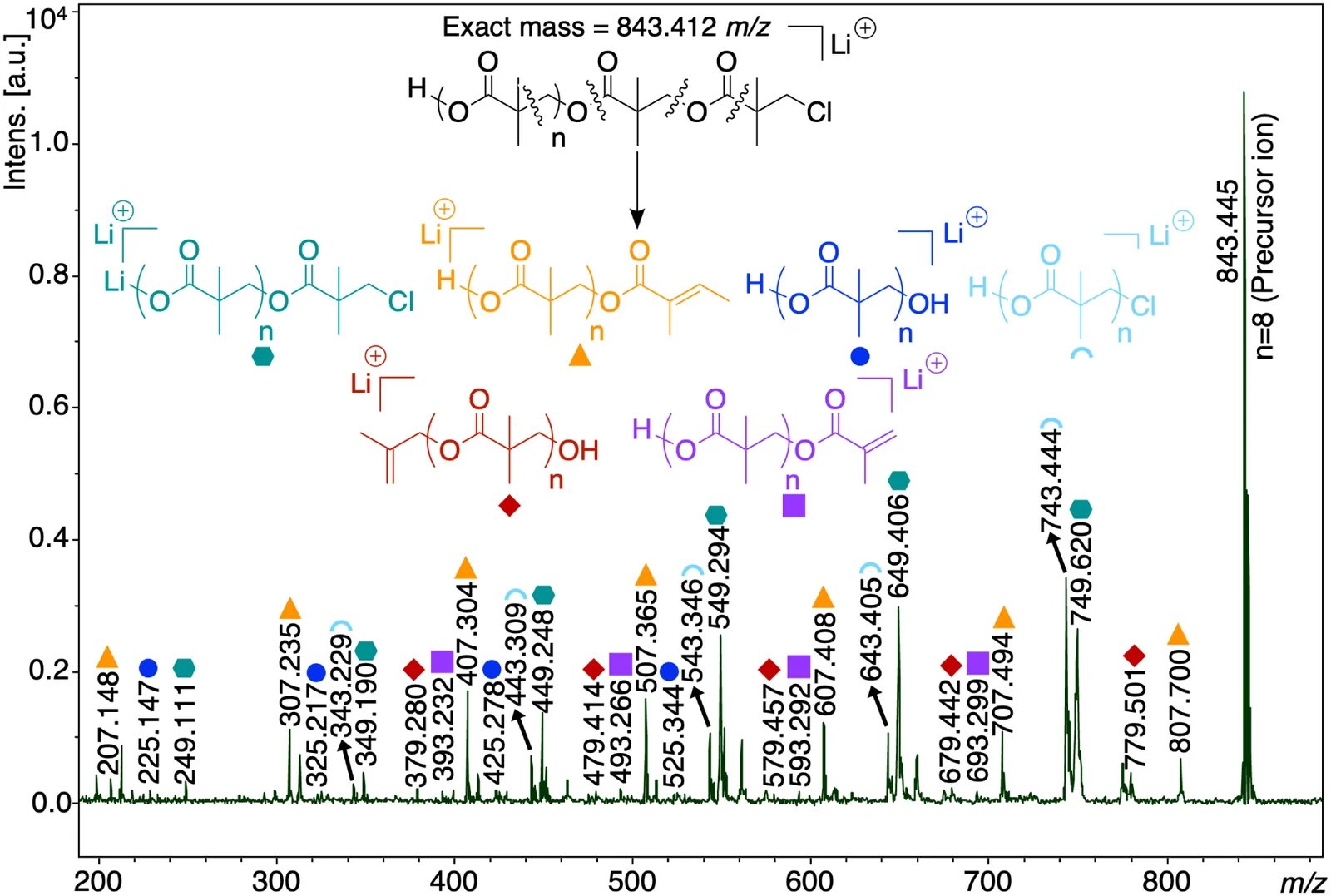
MALDI-ToF MS^2^ mass spectrum of lithiated poly­(DMP)-Cl 8-mer precursor and its six different fragmentation products. The fragments marked with teal hexagons originate from coadmitted (not resolved) dilithiated 8-mer (see text).

The CH_2_—OC=O bond cleavage followed by a 1,2-methyl shift^43^ results in two different products, a Cl-terminated poly­(DMP), shown by light blue arcs, and a 2-methyl-2-butenoate-terminated poly­(DMP), shown by orange triangles (Scheme SI 3A). As seen in the poly­(DMP)–OH spectrum (Figure 1), the replacement of the carboxylic acid proton with a Li^+^ cation is also observed in the Cl-terminated poly­(DMP), represented by teal hexagons. This distribution, formed through the same mechanism (Scheme SI 3A′), is attributed to contamination of the selected lithiated poly­(DMP)-Cl 8-mer with dilithiated 8-mer which is more abundant (Figure SI 6). These two precursor ions differ in mass by only 6 Da and cannot be well resolved under MALDI-ToF MS^2^ conditions, where a TIS is used for precursor ion isolation (vide supra). Meanwhile, the peak observed at *m*/*z* 807.700 in Figure 4 provides evidence that the chloride end group can be eliminated via a 1,2-methyl shift, resulting in the formation of a terminal C=C bond (orange) without the cleavage of a monomer repeat unit.

Cleavage of O—C=O ester bond^48^ eventually yields a water-terminated poly­(DMP) (blue circles), which further fragments to form the red disubstituted alkene (red diamonds) in Figure 4. The mechanism for this fragmentation is shown in Scheme SI 3B. A second possible product, that contains both an alkene and a chlorine end group, was expected via CO loss; however, this is not observed in the spectrum, likely due to its nonpolar nature and competition with more polar products for cation binding (Scheme SI 3B).

It is not unusual for identical fragments to be produced through different mechanisms in MS^2^. An alternative pathway that yields the same products shown in Scheme SI 3B is illustrated in Scheme SI 3C. Cleavage of the OC=O—C­(CH_3_)_2_ bond generates a tertiary alkyl radical ion, which, following the loss of a hydrogen radical, can form a disubstituted alkene (not observed in MS^2^). Subsequent CO loss^47^ from the alkoxycarbonyl radical, accompanied by hydrogen atom transfer from the tertiary radicals, results in the formation of an OH-terminated product (blue circles). The latter product can further fragment to form the disubstituted alkene (red diamonds).

Homolytic cleavage of a carbon–carbon bond is a known,^49^ but not the one next to a quaternary carbon. In Scheme SI 3D, a C–C scission generates a methyl ester radical, which subsequently eliminates formaldehyde and CO to yield the disubstituted alkene product (black), which was not observed in the spectrum. The purple square fragments (Figure 4) arise from hydrogen radical loss and methacrylate end group formation.

Overall, the orange triangle pro duct is dominant from *m*/*z* 200–400 whereas the teal hexagon product is dominant from *m*/*z* 500–800. Both are formed via the mechanism in Scheme SI 3A from lithiated or dilithiated poly­(DMP)-Cl, respectively. Evidently, CH_2_–OCO cleavage followed by a 1,2-methyl shift is the preferred fragmentation pathway for neopentyl polyesters. When Cl is present as the end group, it readily dissociates, promoting the formation of terminal C=C bonds (orange triangles). In contrast, OH is a poor leaving group, and cleavage of C–O bonds in ethers or alcohols requires higher dissociation energies than cleavage of C–Cl bonds, making the orange triangle distribution secondary in linear poly­(DMP)–OH spectra (Figure 1).

### MS^2^ of Poly­(DMP)-Br

Here, a linear poly­(DMP) bearing a bromine atom in place of the terminal hydroxyl group (poly­(DMP)-Br) is investigated. One equivalent of the monomer, 3-bromo-2,2-dimethylpropanoic acid, was reacted with four equivalents of sodium methoxide in the presence of toluene to produce poly­(DMP)-Br. The product was characterized using ^1^H NMR and MALDI-ToF MS.

The MALDI-ToF MS spectrum of linear poly­(DMP)-Br is shown in Figure SI 7. As seen with the chlorine end group, two distinct product distributions are observed. One distribution, labeled as A_n_, corresponds to species in which the proton of the terminal carboxylic acid (COOH) group is intact, and it ionizes with Li^+^ as the cation, whereas the second distribution, denoted by B_n_, has this proton removed and contains the corresponding lithium carboxylate, plus ionizing with a second lithium cation.

Poly­(DMP)-Br was fragmented into five different products as seen for the lithiated 9-mer in Figure 5. The mechanism leading to these products is shown in Scheme SI 4. The characteristic isotopic pattern of one bromine, arising from the nearly equal natural abundances of ^79^Br (50.7%) and ^81^Br (49.3%), has been used to reduce the spectral complexity of fragment ion series.^64,65^ Bromine-containing fragments can be unambiguously identified by their characteristic isotope pattern, manifesting as two very similar peaks at M^+^ and [M+2]^+^.

**Figure 5:**
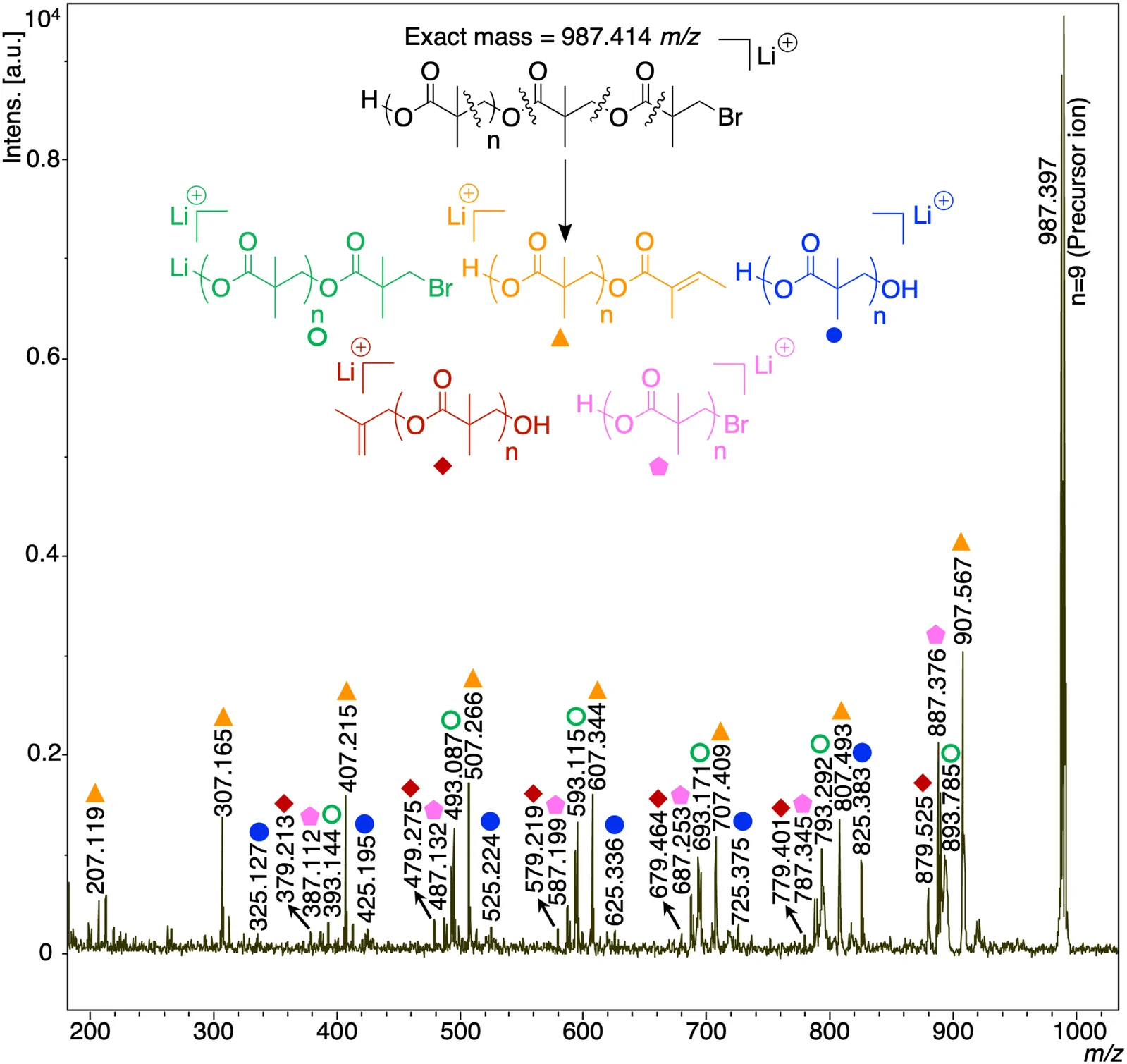
MALDI-ToF MS^2^ mass spectrum of lithiated poly­(DMP)-Br 8-mer precursor and its five different fragmentation products. The fragments marked with green hollow circles originate from coadmitted (not resolved) dilithiated 8-mer (see text).

Cleavage of the CH_2_—OC=O bond, followed by a 1,2-methyl shift,^43^ results in two different products, a Br-terminated poly­(DMP) shown in pink pentagons, and a 2-methyl-2-butenoate-containing poly­(DMP), shown in orange triangles (Figure 5 and Scheme SI 4A). Again, replacement of the carboxylic acid proton with a Li^+^ cation in the Br-terminated poly­(DMP) is observed by green hollow circles in Figure 5. This product must originate from dilithiated poly­(DMP)-Br 8-mer which cannot be resolved from the lithiated 8-mer due to their small mass difference (Figure 5 and Scheme SI 4A′). On the other hand, the peak observed at 907.567 *m*/*z* in Figure 5 provides evidence that the bromide end group can be eliminated via a 1,2-methyl shift, resulting in the formation of a terminal C=C bond (orange triangles) without the cleavage of a monomer repeat unit.

Cleavage of O—C=O ester bond^48^ eventually yields a water-terminated poly­(DMP) (blue circles in Figure 5), which further fragments to form the red disubstituted alkene (red diamonds in Figure 5). The mechanism for this fragmentation is shown in Scheme SI 4B. A second possible product, containing both an alkene and a bromine end group, was expected to arise via CO loss; however, it was not observed in the spectrum, likely due to its nonpolar nature and competition with more polar products for cation binding.

An alternative pathway that yields the same products shown in Scheme SI 4B is illustrated in Scheme SI 4C. Cleavage of the OC=O—C­(CH_3_)_2_ bond generates a tertiary alkyl radical ion, which can transfer or lose a hydrogen radical to form a disubstituted alkene (not observed in MS^2^). Consecutive CO loss^47^ from the other alkoxycarbonyl radical results in the formation of an OH product (blue circles). The OH product can further fragment to form the red-disubstituted alkene (diamonds).

In general, a homolytic cleavage of a carbon–carbon bond is a known phenomenon.^49^ In Scheme SI 4D, a C–C scission generates a methyl ester radical, which later eliminates formaldehyde and CO to yield the disubstituted alkene product (black), which was not seen in the spectrum. The purple square fragment distribution arises from hydrogen radical loss and methacrylate end group formation.

Overall, the orange product is dominant throughout the spectrum, and the same trend was seen in cyclic poly­(DMP). Br as the end group, which readily dissociates, promotes the formation of terminal C=C bonds (orange triangle products). In contrast, OH (Figure 1) and Cl (Figure 5) are poorer leaving groups, and cleavage of C–O and C–Cl bonds requires higher dissociation energies than the C–Br bond, making the orange distribution secondary in linear poly­(DMP)–OH and less competitive in linear poly­(DMP)-Cl spectra.

### MS^2^ of Poly­(DMP)-Pyr

Linear poly­(DMP) with a pyridine initiating group (poly­(DMP)-pyr) was synthesized using a four-membered 3,3-dimethylpropiolactone in bulk. Pyridine initiated the ring opening of the lactone and remained attached to one end of the polymer chain. The MALDI-ToF MS spectrum of poly­(DMP)-pyr is shown in Figure SI 8. Two distinct product distributions were observed. One distribution, marked by A_n_, corresponds to species in which the proton of the terminal carboxylic acid (COOH) group is intact, and it ionizes with Li^+^ as the cation, whereas the second distribution, denoted by B_n_, has this proton removed, and it is replaced as the lithium carboxylate, plus ionizes with a second lithium as the cation.

Figure 6 shows the MS^2^ spectrum of the 8-mer precursor (*m*/*z* 880.460), which fragments into four distinct products. Cleavage at the ester bond leads to an alkoxycarbonyl radical and an acyl radical. The alkoxy radical abstracts a hydrogen atom from the fragment containing an acyl radical to form an OH-terminated fragment (blue circles), whereas the elimination of carbon monoxide from the acyl radical after the loss of a hydrogen radical yields a C=C bond (sky-blue stars) (Scheme SI 5A). The CH_2_—OC=O bond cleavage followed by a 1,2-methyl shift^43^ results in a pyridinium-terminated poly­(DMP) (green crosses) through the elimination of a neutral fragment with a trisubstituted alkene on the end groups (orange triangles) (Scheme SI 5B). The major fragment distribution, indicated by blue circles, corresponds to a water-terminated product formed by cleavage between the O—C=O ester bond^48^ accompanied by a hydrogen atom transfer. This distribution is ionized by Li^+^ which was not included in the selected precursor ion. It is attributed to contamination of the selected precursor with coadmitted poly­(DMP)-pyr 8-mer containing a COOLi chain end (distribution B_n_ in Figure SI 8). This coadmitted contaminant must be the source of the fragments shown by orange triangles as well.

**Figure 6:**
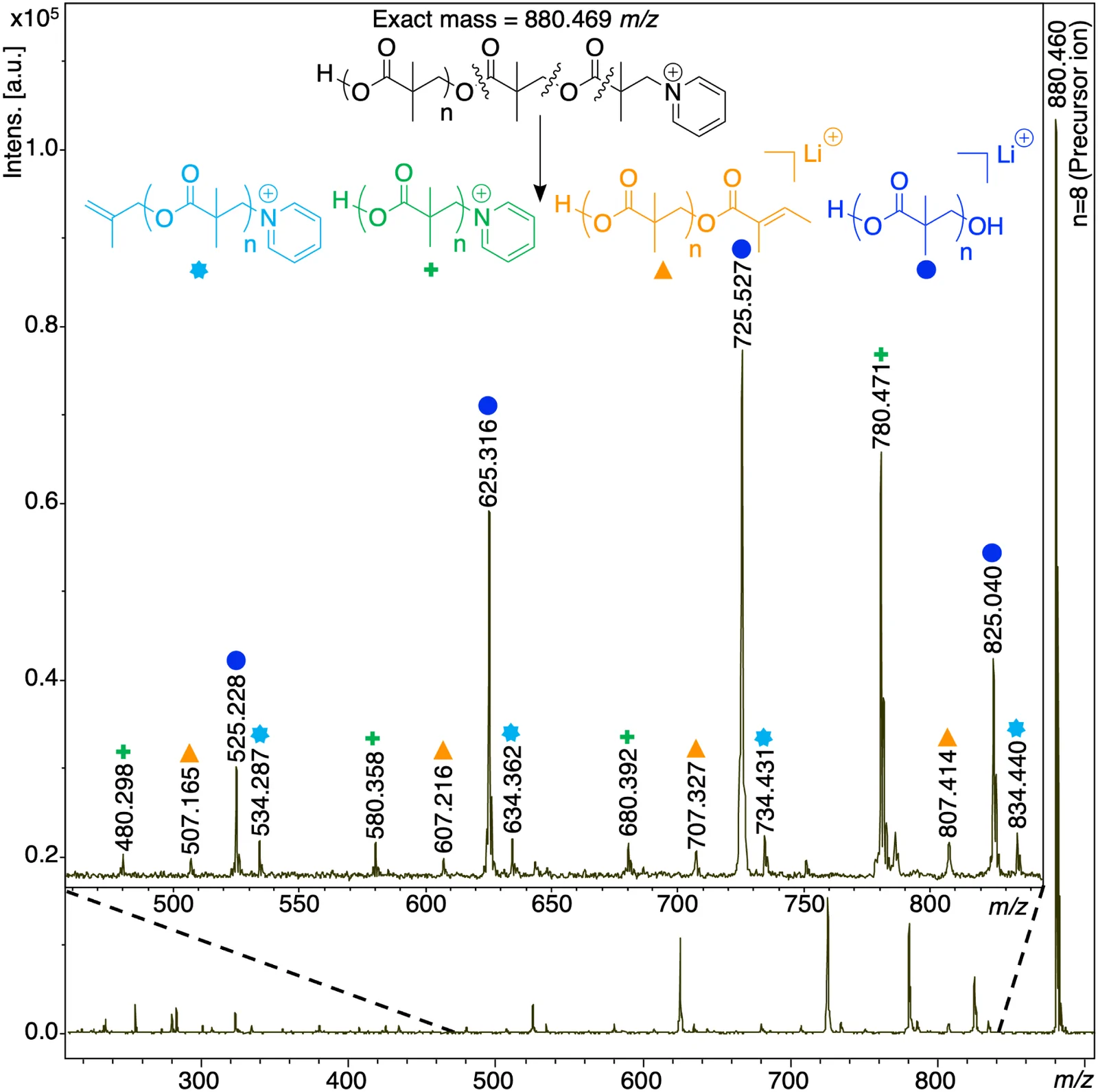
MALDI-ToF MS^2^ mass spectrum of poly­(DMP)-pyr 8-mer and its four different fragmentation products.

The fragments shown by blue circles and orange triangles require ionization by the Li^+^ cation, since they do not carry an inherent charge. In contrast, the distribution shown by light blue stars and green crosses does not need a cation for ionization because the pyridine contains a quaternary nitrogen, which carries a permanent positive charge (Figure SI 9). MALDI-ToF MS^2^ has a very broad isolation window, which allows not only the precursor pyridinium ion (*m*/*z* 880) to pass through but also the pyridinium ion with a COOLi end group (*m*/*z* 886). This effect is amplified by their small-mass difference (6 Da). As Prentice et al. demonstrated, adjusting the timed ion selector window in MS^2^ can achieve tighter precursor isolation, but this comes at the cost of reduced fragment sensitivity.^66^ Therefore, the TIS used in this experiment represents an optimal compromise.

Peaks at *m*/*z* 834.440, *m*/*z* 825.040, and *m*/*z* 807.414 do not include the loss of a monomer. The peak at *m*/*z* 834.440 arises from the cleavage of formic acid, which results in the formation of a double bond on one end and a pyridine group on the other end (sky blue star). The peak at *m*/*z* 825.040 (blue circles) corresponds to the loss of the pyridinium ion from the lithiated contaminant, resulting in a neutral pyridine fragment and a polymer with COOLi and CH_2_^+^ end groups. H_2_O from adventitious moisture produced the peak observed. Finally, the peak at *m*/*z* 807.414 indicates that the pyridinium group cleaves off readily from the lithiated contaminant and is followed by a 1,2-CH_3_ shift that produces an alkene (orange triangle). The fragments marked with blue circles and orange triangles disappear if the samples analyzed are not doped with a Li^+^ salt (Figure SI 9) or are doped with other cations such as Na^+^ and K^+^ (Figure SI 10B and 10C), which have a higher mass difference than Li^+^. This corroborates that those fragments originate from derivatized DMP chains that have undergone a COOH to COOLi exchange.

Pyridinium is a fixed-charge tag where the positive charge is localized and strongly stabilized by the aromatic resonance of the pyridine ring. The quaternary nitrogen also lacks a proton, unlike in peptides, which has been proven to lead the fragmentation via charge-directed pathways.^67^ Therefore, the charge is not mobile, so the fragmentation pathways that retain and stabilize the positive charge on the polymer are generally favored. As a result, charge-remote fragmentation preferentially occurs along the polymer backbone (Scheme SI 5).^68,69^ This explains why the pyridinium-terminated polymer fragments differently compared to the polymer terminated with C–OH, C–Cl, and C–Br.

## Conclusion

The MS^2^ spectra of cyclic and linear poly­(dimethylpropanoate) reveal distinct fragmentation patterns that provide valuable structural insights. Cyclic polymers undergo ring opening as an additional step to generate a mixture of fragments, whereas linear polymers typically fragment via single-bond cleavages. The presence of a neopentyl carbon in poly­(DMP) inhibits the common proton rearrangements observed in other polyesters.

Fragmentation pathways involve cleavage of ester linkages and carbon–carbon bonds, producing various end groups such as water- and halogen-terminated plus pyridinium-initiated fragments. Notably, end group identity influences fragmentation patterns: linear poly­(DMP)–OH predominantly yields water-terminated fragments, poly­(DMP)-Cl favors chlorine-terminated species from *m*/*z* 500–800 and alkene-terminated products from *m*/*z* 200–400, and poly­(DMP)-Br mainly produces alkene-terminated products. This trend is due to a facile 1,2-methyl shift which encourages elimination of the end groups based on their atomic size. Bromine has a comparatively bigger size than chlorine and hydroxyl group which allows it to dissociate more easily. For poly­(DMP)-pyr, pyridinium-terminated ions dominate unless the COOH is replaced by COOLi. The small mass difference of Li^+^ cation led to contamination of the selected precursor ions for the linear polymers. As a result, the proton replacement of the COOH end group with Li (COOLi) was also observed in the fragments.

Cyclic poly­(DMP) underwent ring opening, which produced a C=C on one of the end groups. This linear product further breaks into oligomers with an alkene end group, hence increasing the intensity of alkene-terminated products, unlike the linear counterpart. Ion mobility mass spectrometry further differentiates isomeric fragments by exploiting differences in collision cross-section, distinguishing cyclic precursor ions with smaller CCS from linear fragment ions of identical mass. This emphasizes that the combined use of MS^2^ and IM-MS is essential for accurately resolving polymer structures and distinguishing the isomeric species.

## Experimental Section

### Characterization

A Bruker UltrafleXtreme MALDI-ToF mass spectrometer (Bruker Daltonics, Billerica, MA) was used to collect data. Mass spectral data were collected in positive reflectron ion detection mode. Collision-ion dissociation (CID) was used with Argon being the source of collision gas. The stock solutions were prepared in 1,1,1,3,3,3-hexafluoro-2-propanol to avoid the polymer from precipitating out of solution. The stock solutions of *trans*-2-[3-(4-*tert*-butylphenyl)-2-methyl-2-propenylidene]­malononitrile (DCTB), polymer sample, and lithium trifluoroacetate were prepared in 20 mg/mL, 10 mg/mL, and 10 mg/mL, respectively. The stock solutions were combined in a 20:5:2 ratio (v/v) (matrix/analyte/cation) for all the samples. However, for poly­(DMP)–OH, a 20:5:5 ratio (v/v) was used as it gave a better signal-to-noise ratio. 0.5 μL was plated via the dried droplet method. MALDI-ToF MS spectra were calibrated against SpheriCal dendritic calibrants (Polymer Factory, Sweden). The mass accuracy for fragment ions for all the samples is typically within ±0.05 *m*/*z* but can increase to ±0.2 *m*/*z* for low-intensity fragments near the noise level. The mass accuracies observed for the fragment ions in this study are consistent with these expected values.

All ESI and IM-MS experiments were conducted using a Bruker timsToF Pro 2 (Billerica, MA) equipped with an electrospray ionization source with an average mass resolving power of 50,000 fwhm (full width at half-maximum). The mass range probed was *m*/*z* 200–2000, and the polarity of the timsToF was set to positive mode. For MS^2^ analysis, N_2_ served as the collision gas, with the gas flow rate adjusted at 65%. The precursor ions for MS^2^ experiments were selected with an isolation width of ±3 *m*/*z*, and the collision voltage was set between 10 and 50 eV, depending on the ion of interest. The poly­(DMP) samples were dissolved in either methanol or acetonitrile and then filtered using an Acrodisc 13 mm 0.2 μm PTFE filter before being diluted to concentrations between 20 ng/mL and 10 μg/mL. The samples were then infused into the instrument via a 1 mL syringe. The end plate offset, capillary voltage, nebulizer gas pressure, dry gas flow, and dry gas temperature were set to 400 V, 4000 V, 1.5 bar, 4 L/min, and 300 °C, respectively.

IM-MS experiments were carried out after calibration of the mobility scale (1/*K*_0_) with the Agilent tune mix. Calibration of the instrument was conducted after each experiment. The timsToF software automatically converts 1/*K*_0_ to collision cross-section (CCS) values. The reported CCS data were derived from mobilograms (CCS distributions) collected in triplicate with the voltage between the entrance channel and the analyzer section of the TIMS device (Δ6). Unfolding in the TIMS cell was conducted by increasing the Δ6 DC voltage between 30 and 200 V. Due to the higher energy required to induce unfolding of polymers compared to proteins, Tunnel-in pressure was set below 1.5 mbar. The ramp time and accumulation time were set to 200 and 100 ms, with the mobility scan range being set to 1/*K*_0_ = 0.67–1.56 V·s/cm^2^. The average centroid of each fitted mobility peak was used in the calculation of CCSs, all of which correspond to experimental ^TIMS^CCS_N2_.

## Supplementary Material



## Notes

Additional MALDI-ToF mass spectra of precursor ions, ESI-MS^2^ plus ion mobility mass spectrometry data, and proposed fragmentation mechanisms for linear and cyclic poly­(DMP), including halide-terminated and pyridinium-initiated polymers, as well as poly­(DMP)-pyr tandem MS salt comparison studies (PDF)
