# Supplementary material for: Elucidating the Fragmentation Pathways of Cyclic and Linear Poly(dimethylpropanoate) via Tandem Mass Spectrometry and Ion Mobility Mass Spectrometry
Source: J Am Soc Mass Spectrom. 2026 Oct 23;37(11):2697–711. doi: 10.1021/jasms.6c00172 (PMC13741902; doi:10.1021/jasms.6c00172)
Supplement: Supplementary file 1 [file js6c00172_si_001.pdf]

Elucidating Fragmentation Pathways of Cyclic and Linear Poly(dimethylpropanoate) via Tandem Mass Spectrometry and Ion Mobility Mass Spectrometry

Supporting Information

Amman Nadeem[a], Calum Bochenek[b], Luciana R. Molina[b], Chrys Wesdemiotis[b*], and Scott M. Grayson[a*]

[a]Department of Chemistry, Tulane University, New Orleans, Louisiana, 70118, United States
[b]Department of Polymer Science and Chemistry, The University of Akron, Akron, Ohio, 44325, United States

Corresponding Authors

Scott M. Grayson* - *Department of Chemistry, Percival Stern Hall, Tulane University, New Orleans, Louisiana 70118, United States*.

Email: sgrayson@tulane.edu

Chrys Wesdemiotis* - *Department of Polymer Science and Chemistry, The University of Akron, Akron, Ohio, 44325, United States*

Email: wesdemiotis@uakron.edu

Table of Contents

Scheme SI 1. Reaction scheme for the formation of linear poly(DMP)-OH and cyclic poly(DMP)........SI4

Figure SI 1. MALDI-ToF MS of the linear poly(DMP)-OH precursor........SI5

Figure SI 2. MALDI-ToF MS of the cyclic poly(DMP) precursor........SI6

Scheme SI 2. Fragmentation mechanism of the cyclic poly(DMP)........SI7

Figure SI 3. Electrospray ionization-MS and ion mobility-mass spectrometry (IM-MS) of an 8-mer linear poly(DMP)........S9

Figure SI 4. Electrospray ionization-MS and ion mobility-mass spectrometry (IM-MS) of a 7-mer cyclic poly(DMP)........SI10

Figure SI 5. Electrospray ionization-MS and ion mobility-mass spectrometry (IM-MS) of a 9-mer cyclic poly(DMP)........SI12

Figure SI 6. MALDI-ToF MS of the linear poly(DMP)-Cl precursor........SI14

Scheme SI 3. Fragmentation mechanism of the linear poly(DMP)-Cl........SI15

Figure SI 7. MALDI-ToF MS of the linear poly(DMP)-Br precursor........SI17

Scheme SI 4. Fragmentation mechanism of the linear poly(DMP)-Br........SI18

Figure SI 8. MALDI-ToF MS of the linear poly(DMP)-pyr precursor........SI20

Scheme SI 5. Fragmentation mechanism of the linear poly(DMP)-pyr........SI21

Figure SI 9. Tandem mass spectrometry of 10-mer poly(DMP)-pyr; a comparison between the samples with and without lithium trifluoroacetate salt……………………………………………………..……SI22

Figure SI 10. Tandem mass spectrometry of 8-mer poly(DMP)-pyr; a comparison between the samples with Li^+, Na^+, and K^+ cations………………………………………………………………………………SI23

Experimental Section

Materials…….……SI24

Synthesis of 3-bromo-2,2-dimethylpropanoic acid……………………………………………………………..SI24

Synthesis of 3,3-dimethylpropiolactone. ……………………………………………………………………………SI25

Synthesis of linear Poly(DMP)-OH. ………………………………………………………………………..……………SI25

Synthesis of cyclic Poly(DMP)…………………………………………………………………………………………….SI26

Synthesis of linear Poly(DMP)-Cl.…………………………………………………………………………………………SI26

Synthesis of linear Poly(DMP)-Br.………………………………………………………………………………..………SI27

Synthesis of linear Poly(DMP)-pyr.………………………………………………………………………………………SI27

References……….……SI28

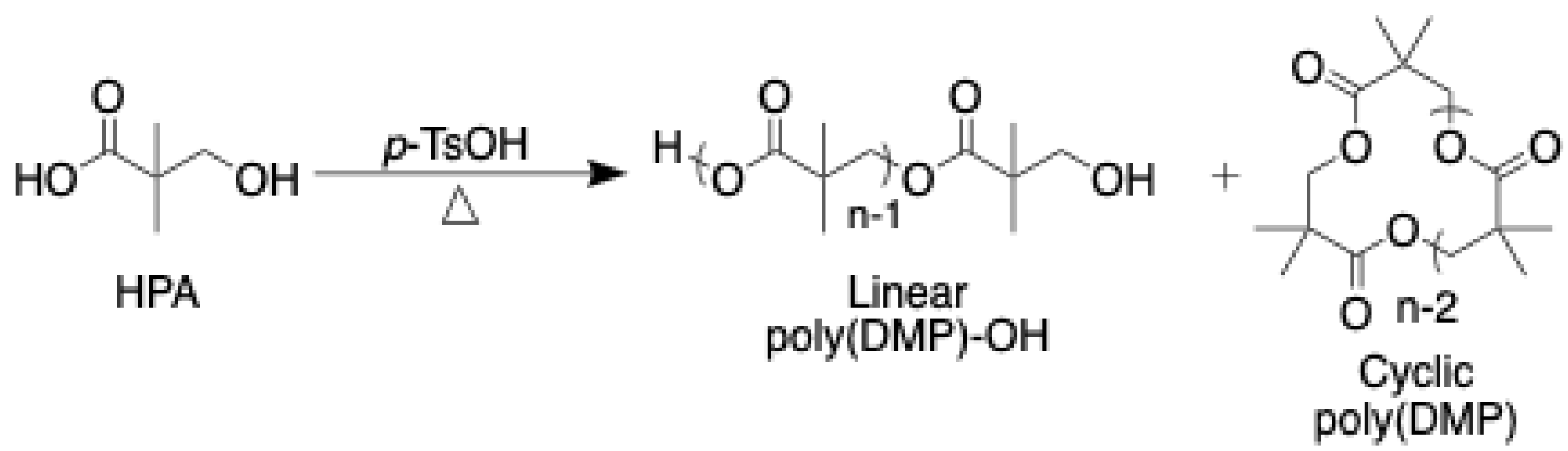

Scheme SI 1. Reaction scheme for the formation of linear poly(DMP)-OH and cyclic poly(DMP). A catalytic amount of *p*-TsOH was used while the temperature was varied from 140 ^{o}C to 230 ^{o}C to control the amount of cyclic formed.

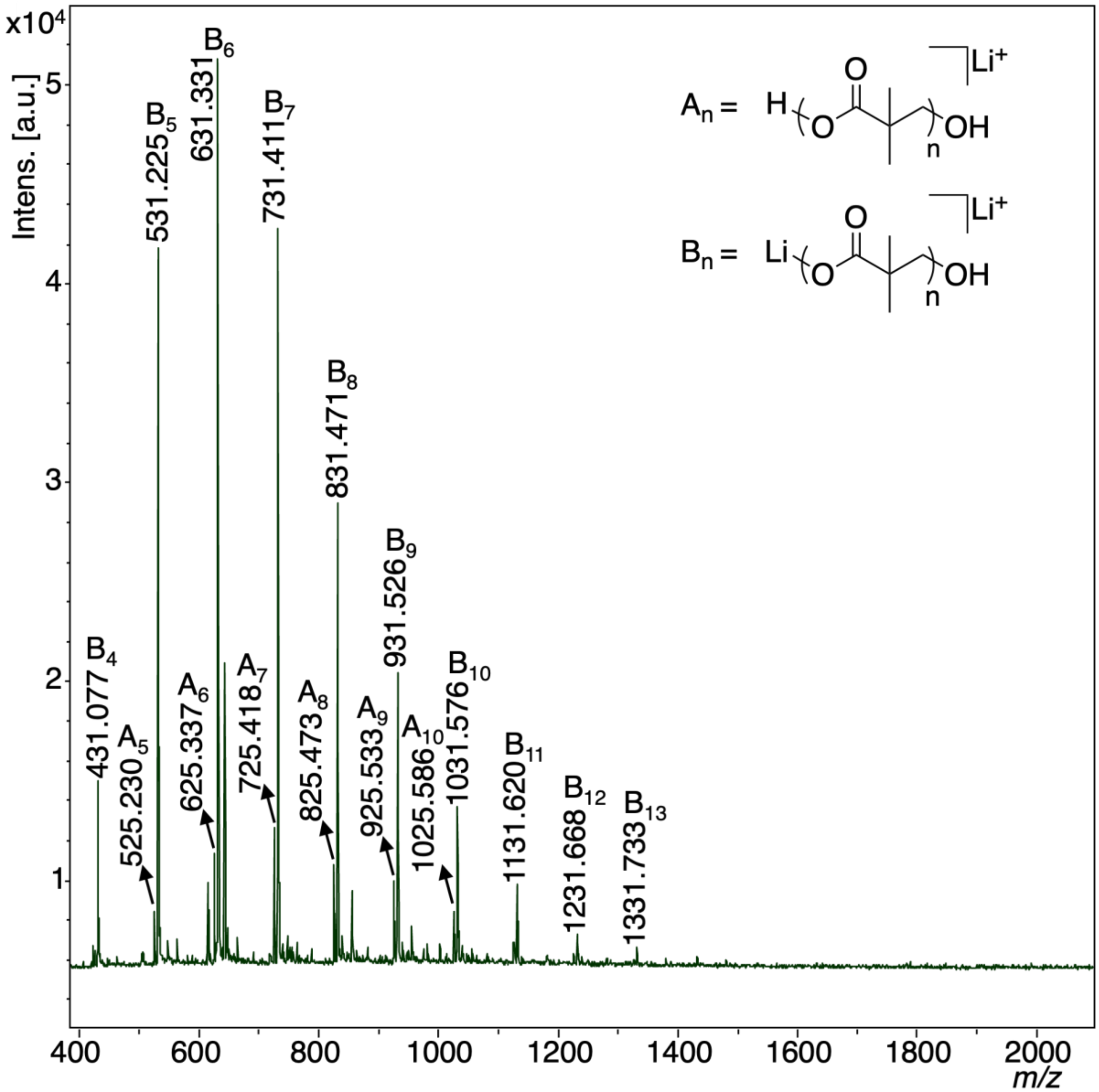

Figure SI 1. MALDI-ToF MS of the linear poly(DMP)-OH, repeat unit = 100.0524 Da ($C_5H_8O_2$), with lithium trifluoroacetate used as a cation source. A_n distribution corresponds to species in which the proton of the terminal carboxylic acid (COOH) group is intact, and it ionizes with Li^+ as the cation. B_n is the primary distribution where the product has proton removed and contains the corresponding lithium carboxylate, plus ionizes with a second lithium cation. "n" represents the number of repeat units.

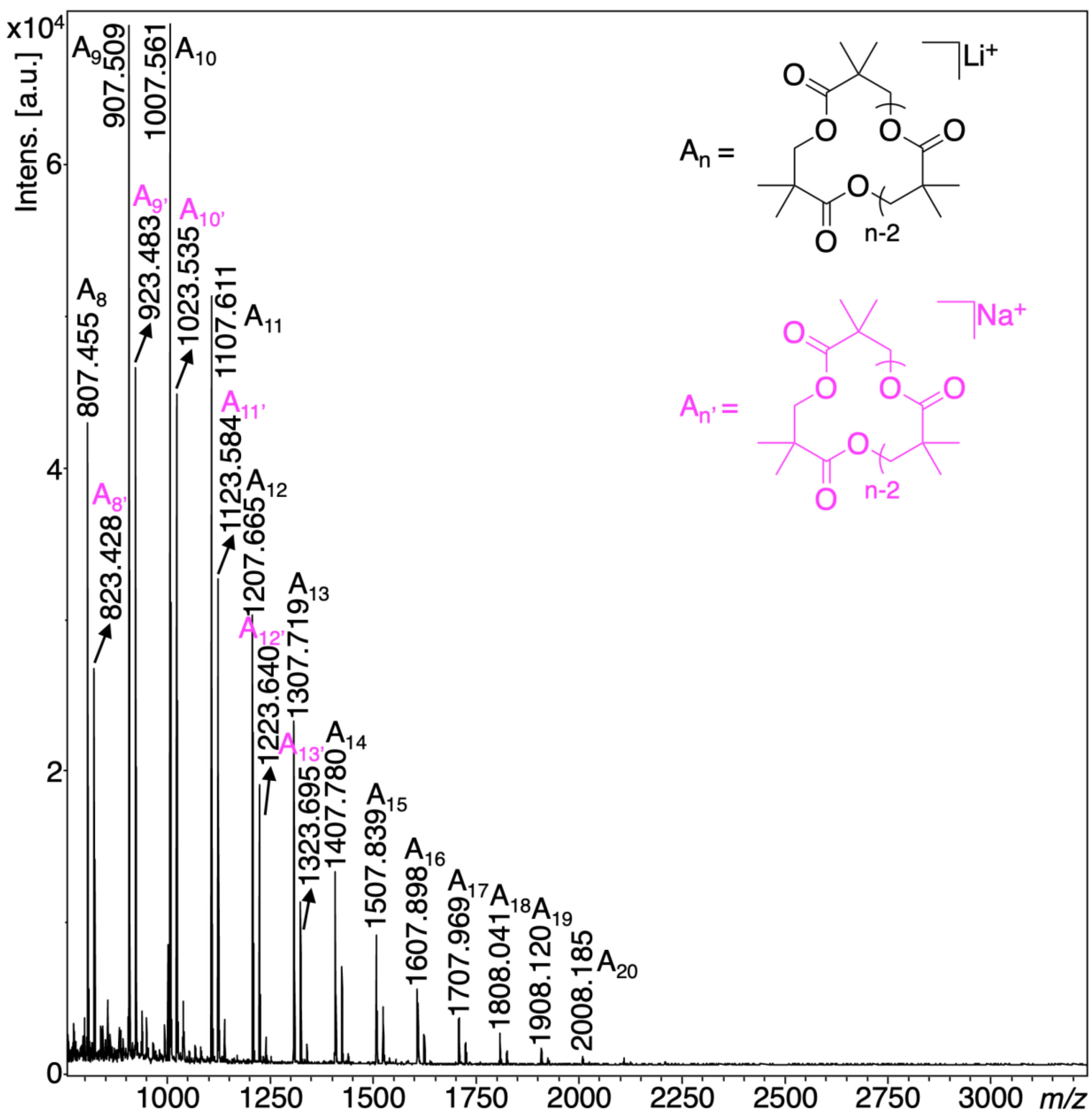

Figure SI 2. MALDI-ToF MS of the cyclic poly(DMP), repeat unit = 100.0524 Da ($C_5H_8O_2$), with lithium trifluoroacetate used as a cation source. A_n is the primary distribution which corresponds to species ionizing with Li^+ as the cation. $A_{n'}$, in pink, represents the distribution where the polymer ionizes with Na^+ as the cation. “n” represents the number of repeat units.

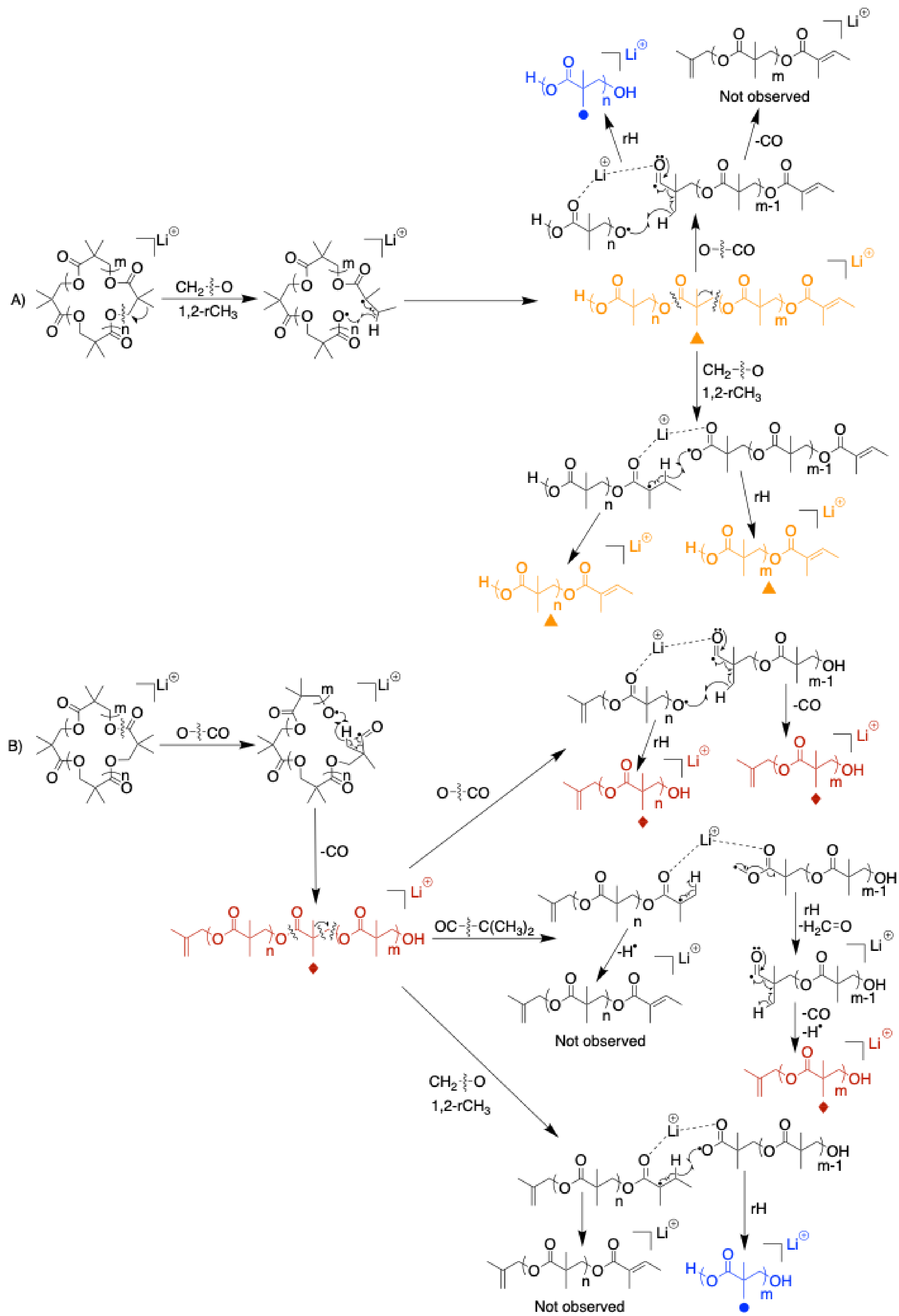
A)
B)
CH2-O
1,2-rCH3
O-CO
OC-C(CH3)2
rH
-CO
rH
-H2C=O
-CO
-H•
-H•
Not observed
Not observed
Not observed

Scheme SI 2. Fragmentation mechanism of the cyclic poly(DMP), where n and m denote the number of repeat units. Cyclic poly(DMP) breaks into a linear chain. The linear chain then fragments further into various oligomers, highlighted in orange triangles, blue circles, black, and red diamonds. Radical-induced fragment dissociation pathways leading to products, where 1,2-rCH_3 and rH designate a 1,2-methyl shift and a proton transfer respectively. There are two bond cleavage pathways, A) cleavage of the methylene C-O bond and B) cleavage of the ester linkage. The orange triangles alkene product can be produced as *E* or *Z* isomer, however, the one shown is more favorable.

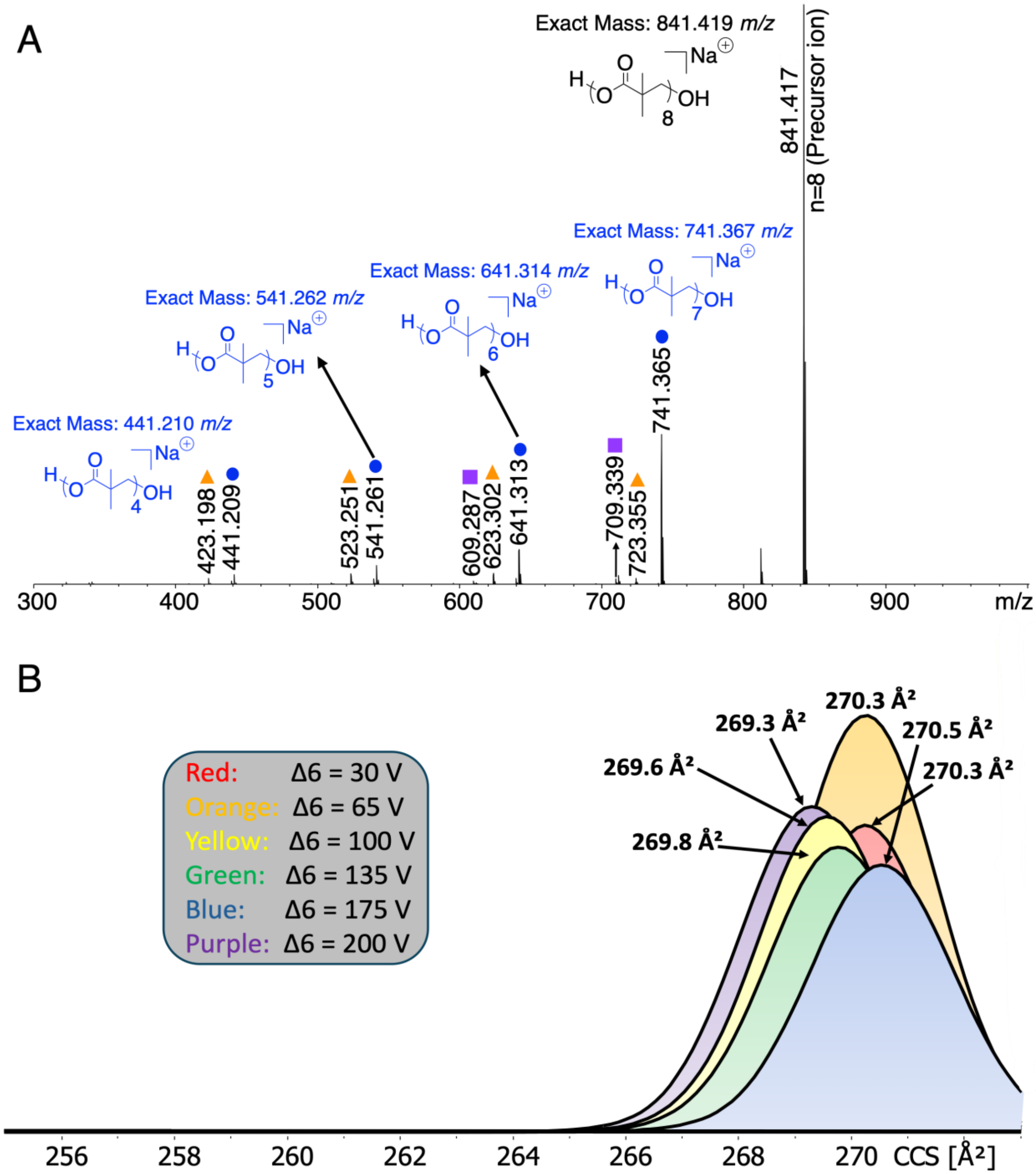

Figure SI 3. (A) Electrospray ionization-MS^2 (ESI-MS^2) spectrum of the 8-mer linear poly(DMP) with sodium cation and (B) Ion mobility-mass spectrometry (IM-MS) spectrum of this 8-mer. The CCS does not change as the voltage increases confirming the linear specie.

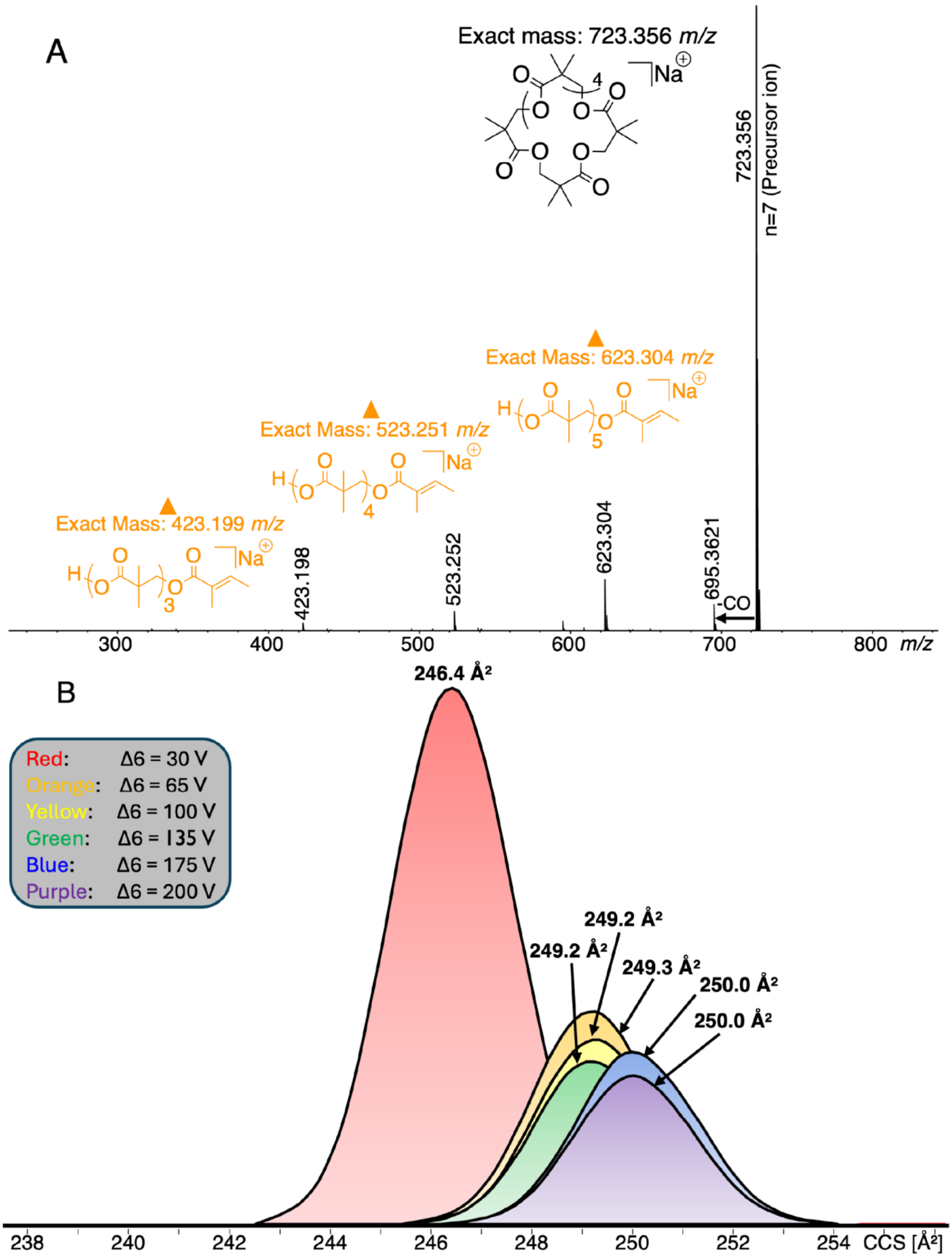

A
Exact mass: 723.356 m/z
Na
723.356
n=7 (Precursor ion)
Exact Mass: 623.304 m/z
Exact Mass: 523.251 m/z
Exact Mass: 423.199 m/z
623.304
523.252
423.198
695.3621
-CO
300
400
500
600
700
800 m/z
B
246.4 Å²
Red: Δ6 = 30 V
Orange: Δ6 = 65 V
Yellow: Δ6 = 100 V
Green: Δ6 = 135 V
Blue: Δ6 = 175 V
Purple: Δ6 = 200 V
249.2 Å²
249.2 Å²
249.3 Å²
250.0 Å²
250.0 Å²
238
240
242
244
246
248
250
252
254
CCS [Å²]

Figure SI 4. (A) Electrospray ionization-MS^2 (ESI-MS^2) spectrum of the 7-mer cyclic poly(DMP) with sodium cation and (B) Ion mobility-mass spectrometry (IM-MS) spectrum of this 7-mer. The CCS increases as the voltage increases indicating ring-opening bond cleavage and the cyclic turning into a linear species.

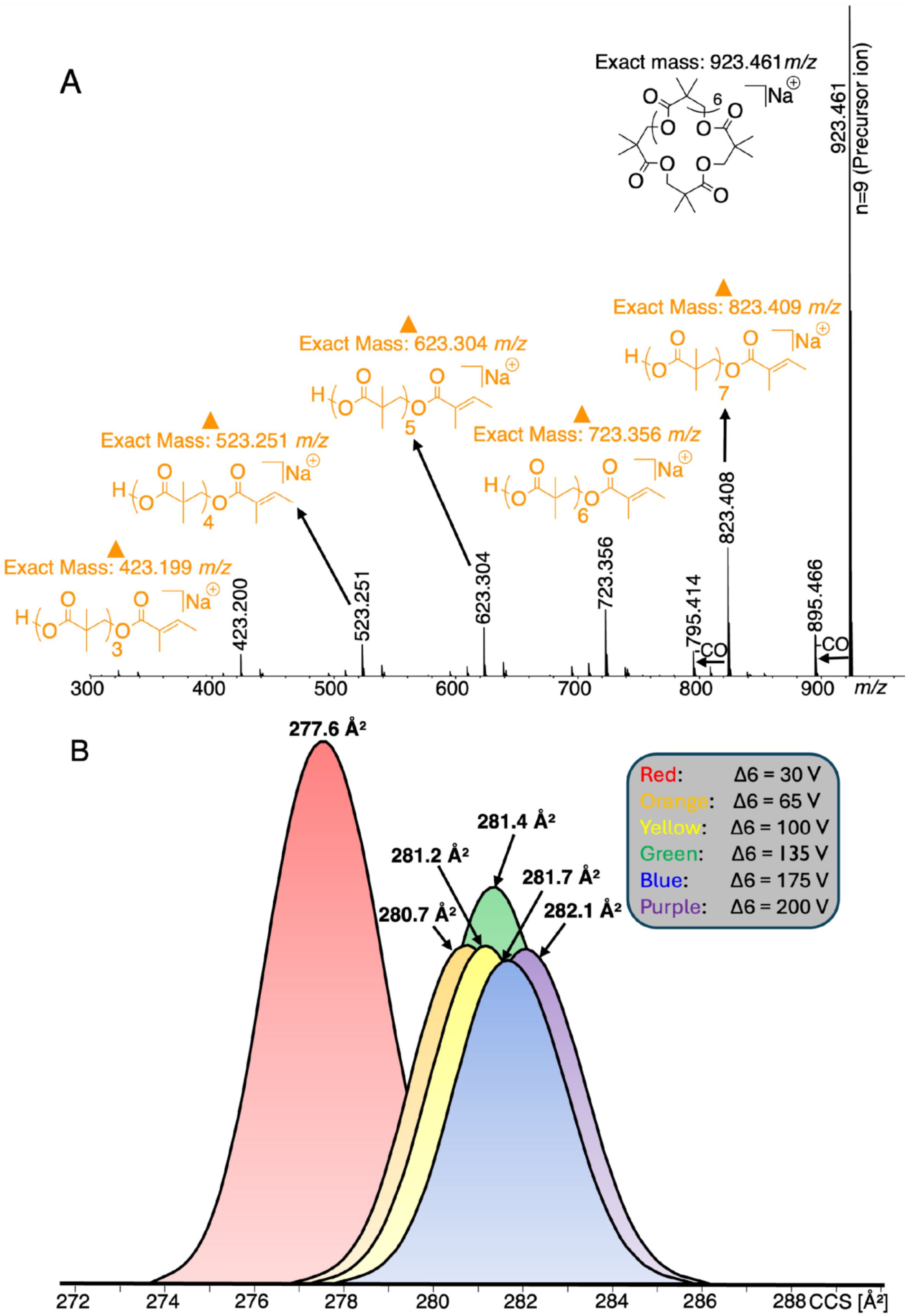
A
Exact mass: 923.461 m/z
Na
923.461
n=9 (Precursor ion)
Exact Mass: 823.409 m/z
Exact Mass: 723.356 m/z
Exact Mass: 623.304 m/z
Exact Mass: 523.251 m/z
Exact Mass: 423.199 m/z
423.200
523.251
623.304
723.356
795.414
823.408
895.466
-CO
m/z
B
277.6 Å²
281.4 Å²
281.2 Å²
281.7 Å²
280.7 Å²
282.1 Å²
Red: Δ6 = 30 V
Orange: Δ6 = 65 V
Yellow: Δ6 = 100 V
Green: Δ6 = 135 V
Blue: Δ6 = 175 V
Purple: Δ6 = 200 V
CCS [Å²]

Figure SI 5. (A) Electrospray ionization-MS^2 (ESI-MS^2) spectrum of the 9-mer cyclic poly(DMP) with sodium cation and (B) Ion mobility-mass spectrometry (IM-MS) spectrum of this 9-mer. The CCS increases as the voltage increases indicating ring-opening bond cleavage and the cyclic turning into a linear species.

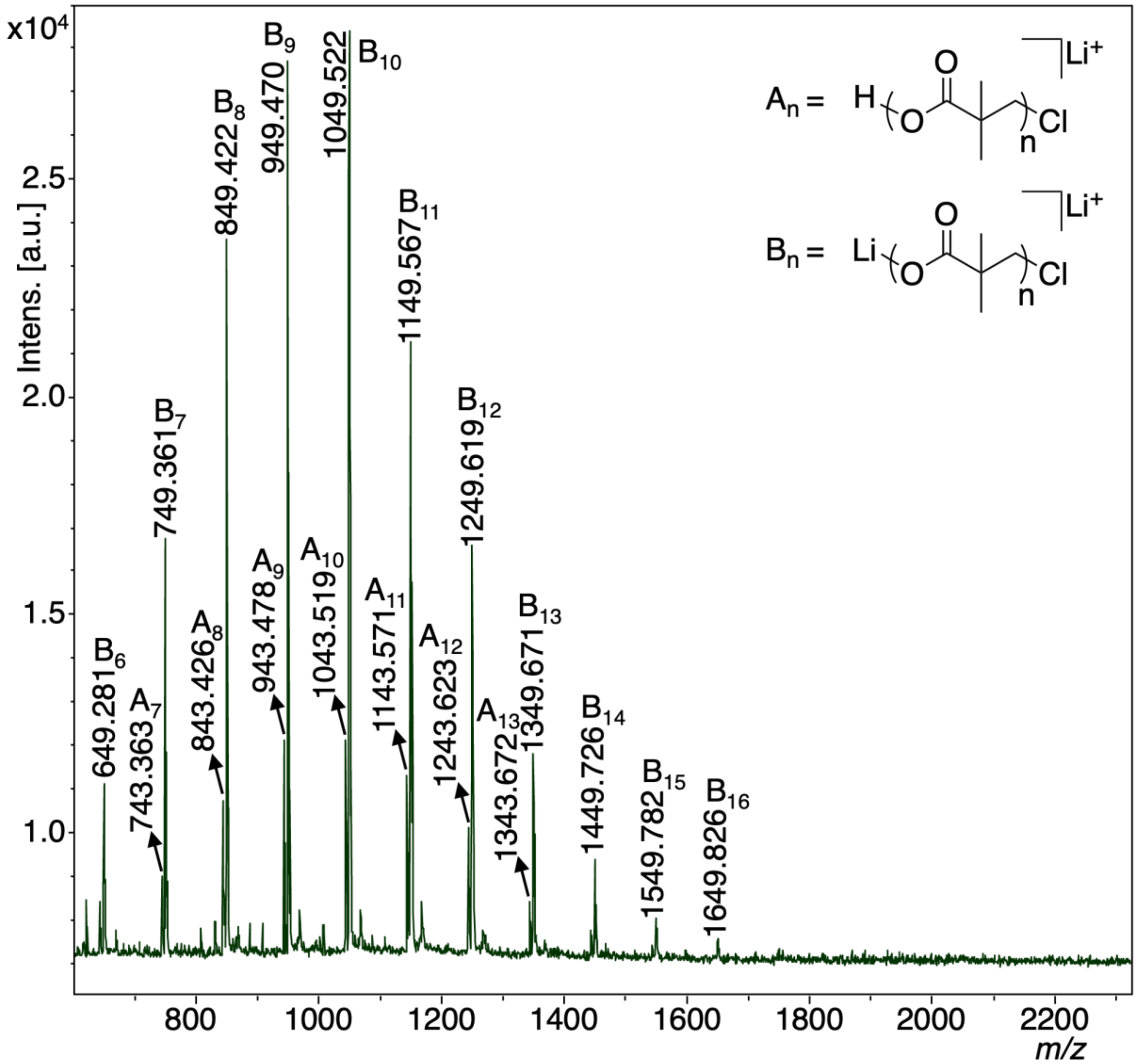

Figure SI 6. MALDI-ToF MS of the linear poly(DMP)-Cl, repeat unit = 100.0524 Da ($C_5H_8O_2$), with lithium trifluoroacetate used as a cation source. A_n distribution corresponds to species in which the proton of the terminal carboxylic acid (COOH) group is intact, and it ionizes with Li^+ as the cation. B_n is the primary distribution where the product has proton removed and contains the corresponding lithium carboxylate, plus ionizes with a second lithium cation. "n" represents the number of repeat units.

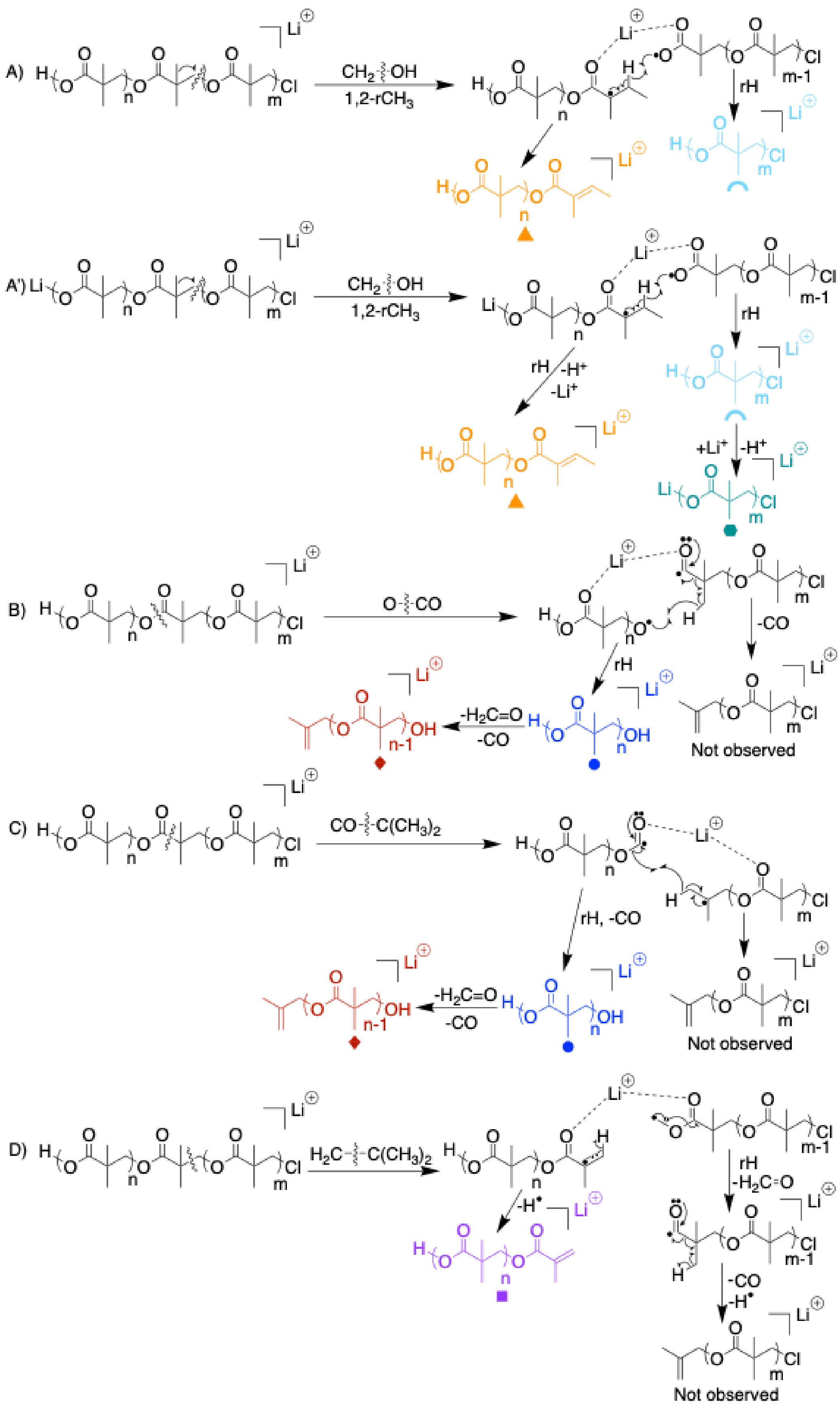
A)
CH2 OH
1,2-rCH3
rH
A')
CH2 OH
1,2-rCH3
rH
rH -H+ -Li+
+Li+ -H+
B)
O CO
-CO
rH
-H2C=O
-CO
Not observed
C)
CO C(CH3)2
rH, -CO
-H2C=O
-CO
Not observed
D)
H2C C(CH3)2
-H•
rH
-H2C=O
-CO
-H•
Not observed

Scheme SI 3. Fragmentation mechanism of the linear poly(DMP)-Cl, where n and m denote the number of repeat units. Radical-induced fragment dissociation pathways leading to products, where 1,2-rCH_3 and rH designate a 1,2-methyl shift and a hydrogen atom transfer respectively. Different bond cleavages result in the formation of distinct fragment ions. A) Cleavage of the methylene C-O bond in the precursor poly(DMP)-Cl ion, A') cleavage of the methylene C-O bond in the poly(DMP)-Cl ion contaminated by Li^+, B) cleavage of the ester linkage, C) cleavage of the bond between carbonyl carbon and the quaternary carbon, and D) cleavage of the bond between methylene carbon and the quaternary carbon. The orange alkene product (triangles) can be produced as *E* or *Z* isomer, however, the one shown is more favorable.

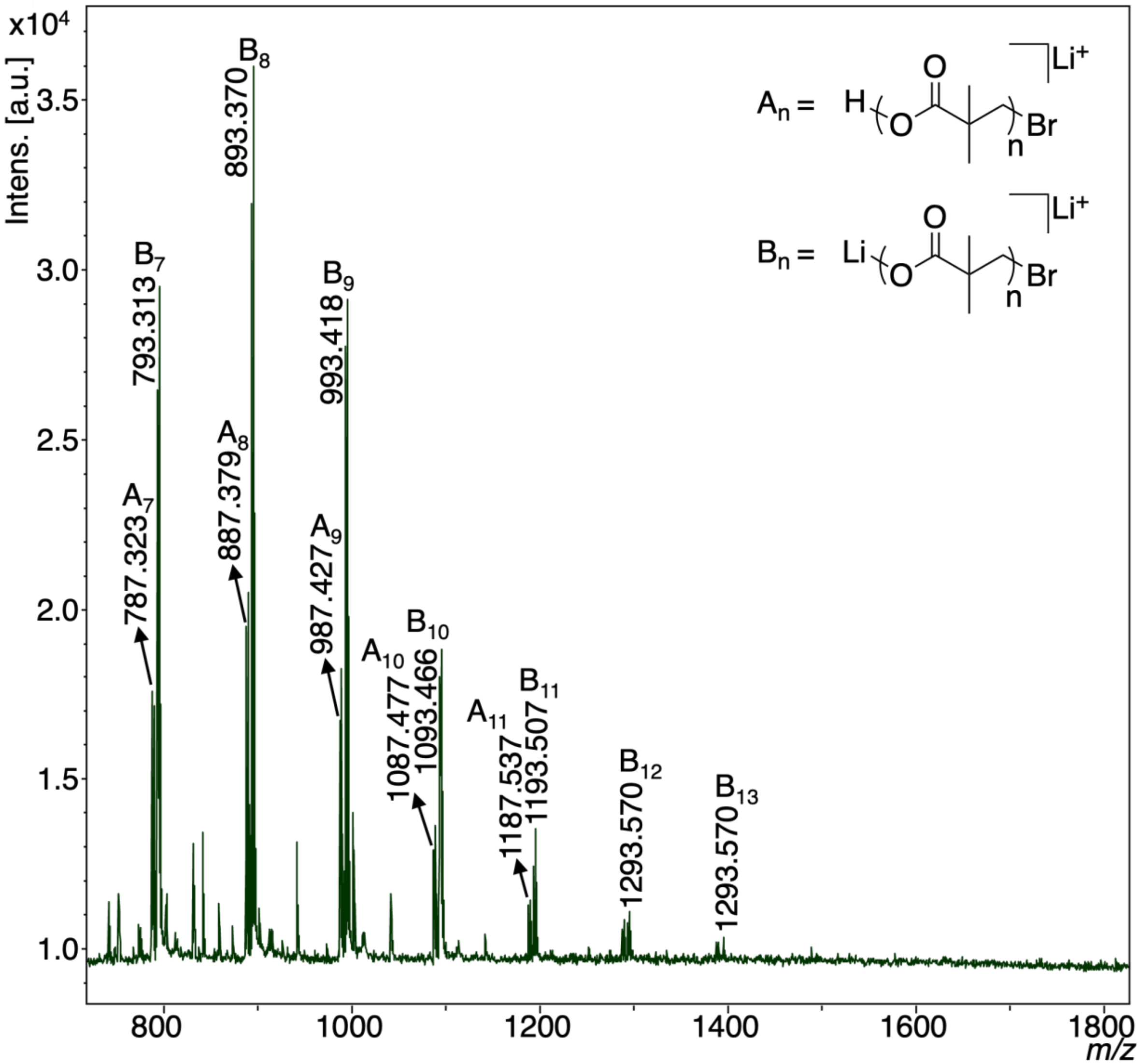

Figure SI 7. MALDI-ToF MS of the linear poly(DMP)-Br, repeat unit = 100.0524 Da ($C_5H_8O_2$), with lithium trifluoroacetate used as a cation source. A_n distribution corresponds to species in which the proton of the terminal carboxylic acid (COOH) group is intact, and it ionizes with Li^+ as the cation. B_n is the primary distribution where the product has proton removed and contains the corresponding lithium carboxylate, plus ionizes with a second lithium cation. “n” represents the number of repeat units.

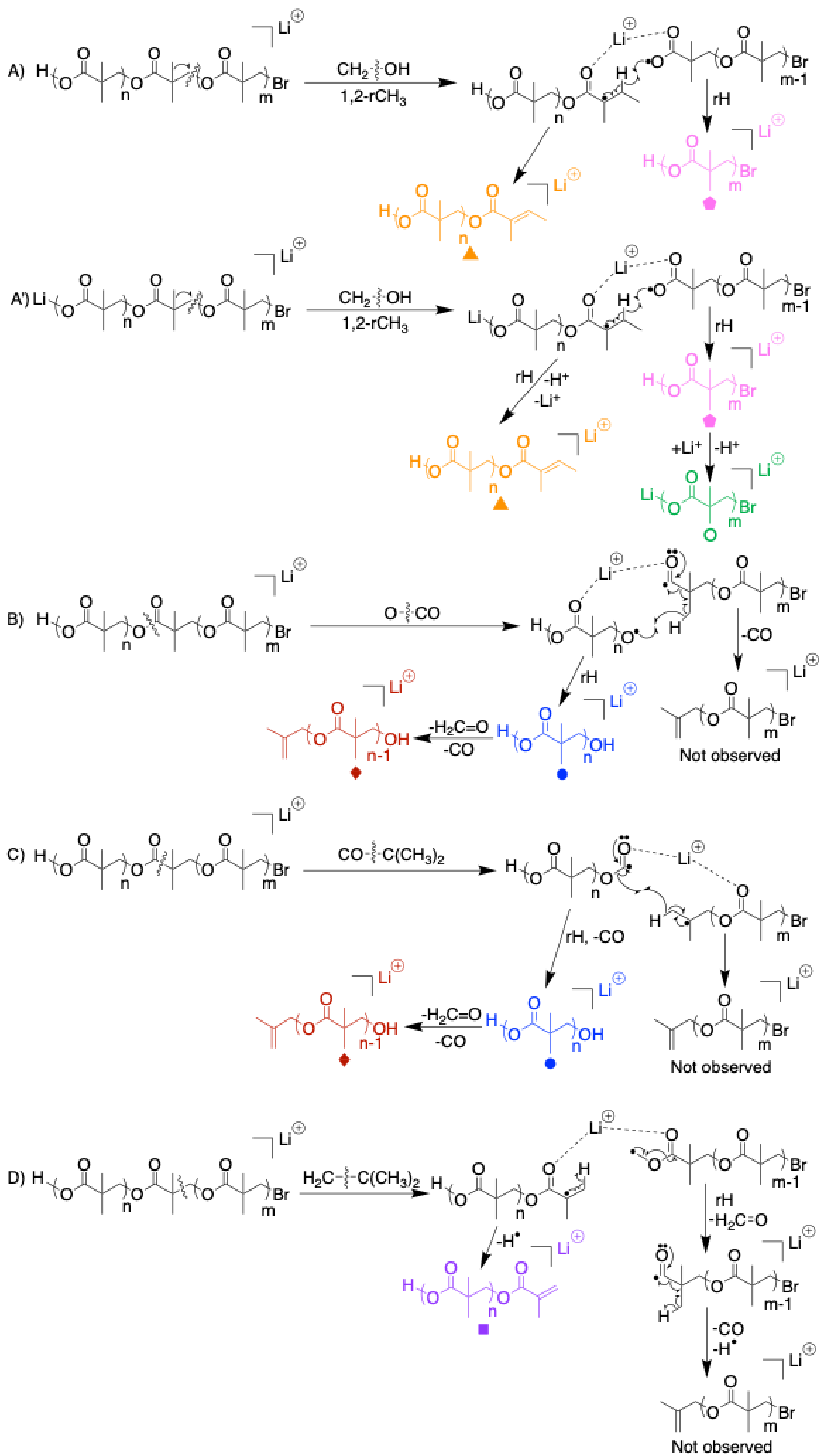

A)
A')
B)
C)
D)
CH2-OH
1,2-rCH3
rH
rH -H+ -Li+
+Li+ -H+
O-CO
-CO
-H2C=O
-CO
CO-C(CH3)2
rH, -CO
H2C-C(CH3)2
-H•
rH
-H2C=O
-CO
-H•
Not observed
Not observed
Not observed

Scheme SI 4. Fragmentation mechanism of the linear poly(DMP)-Br, where n and m denote the number of repeat units. Radical-induced fragment dissociation pathways leading to products, where 1,2-rCH_3 and rH designate a 1,2-methyl shift and a hydrogen atom transfer respectively. Different bond cleavages result in the formation of distinct fragment ions. A) Cleavage of the methylene C-O bond in the precursor poly(DMP)-Br ion, A') cleavage of the methylene C-O bond in the poly(DMP)-Br ion contaminated by Li^+, B) cleavage of the ester linkage, C) cleavage of the bond between carbonyl carbon and the quaternary carbon, and D) cleavage of the bond between methylene carbon and the quaternary carbon. The orange alkene product (triangles) can be produced as *E* or *Z* isomer, however, the one shown is more favorable.

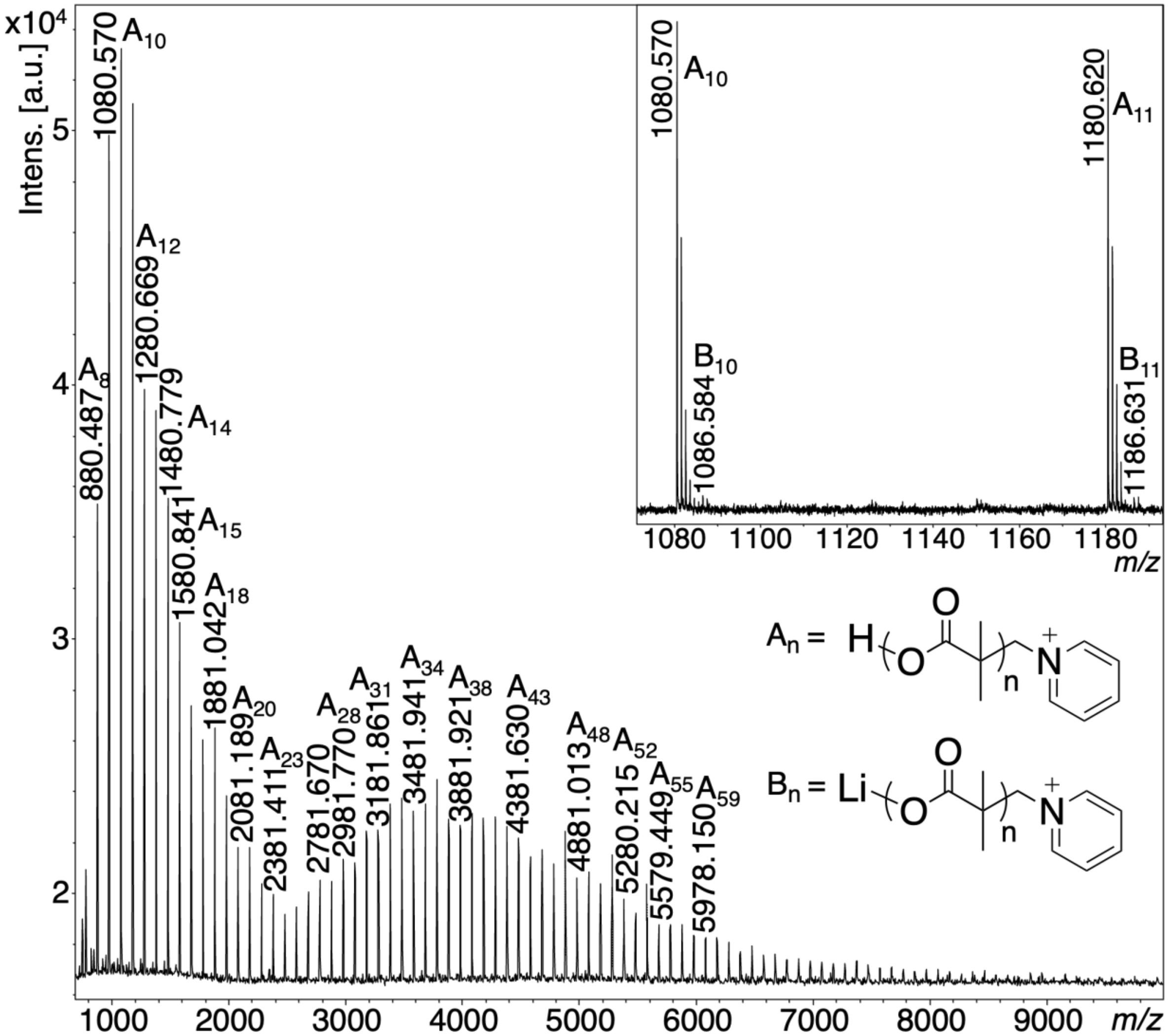

Figure SI 8. MALDI-ToF MS spectrum of the linear poly(DMP)-pyr, repeat unit = 100.0524 Da ($C_5H_8O_2$), with lithium trifluoroacetate used as a cation source. However, the polymer ionizes without a cation because the pyridinium end is positively charged. A_n distribution corresponds to species in which the proton of the terminal carboxylic acid (COOH) group is intact. B_n is the distribution where the product has proton removed and contains the corresponding lithium carboxylate. “n” represents the number of repeat units.

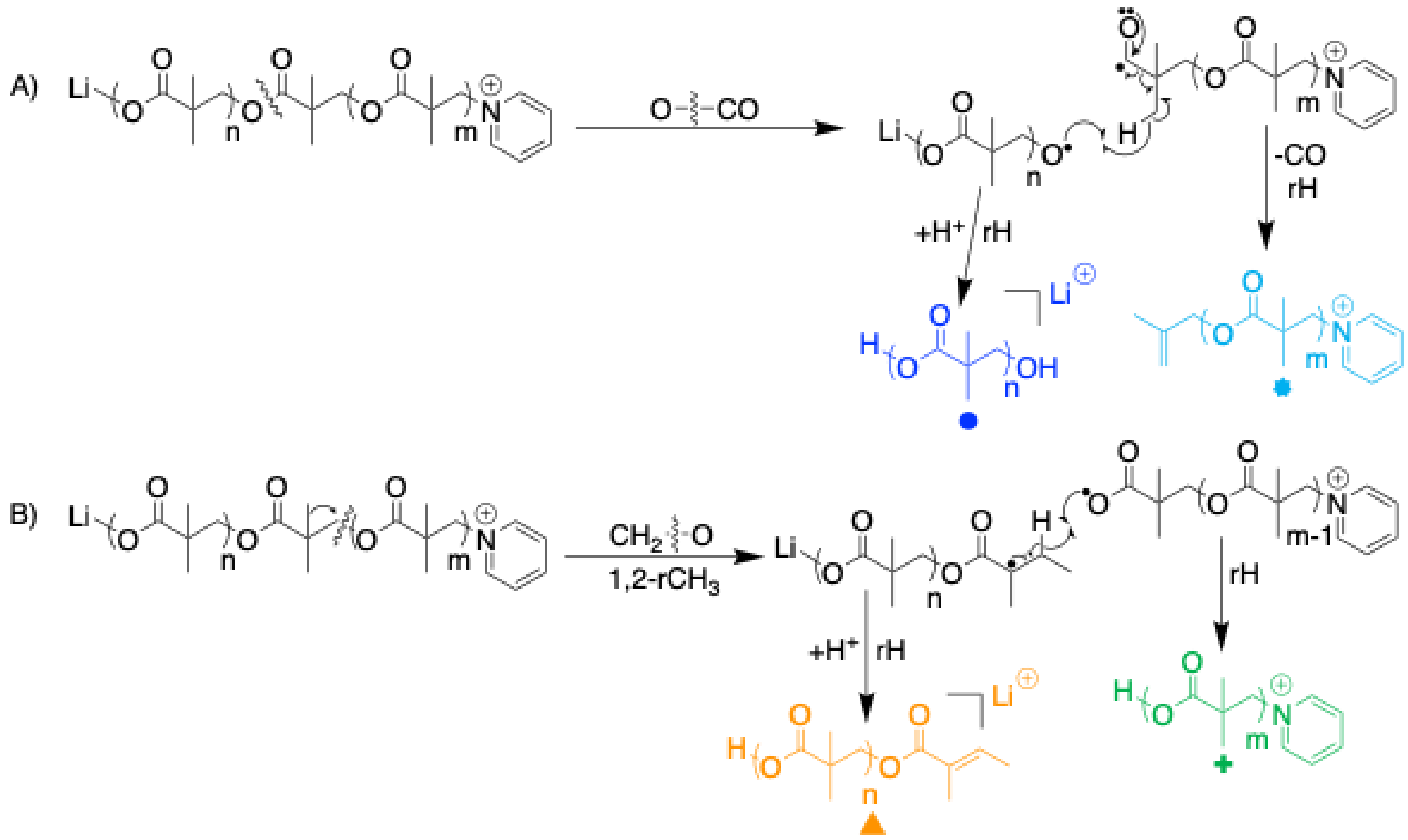

Scheme SI 5. Fragmentation mechanism of poly(DMP)-pyr, contaminated with Li^+ counterion, where n and m denote the number of repeat units. Charge-remote radical-induced fragment dissociation pathways leading to products, where 1,2-rCH_3 and rH designate a 1,2-methyl shift and a proton transfer respectively. There are two bond cleavage pathways, A) cleavage of the ester linkage and B) cleavage of the methylene C-O bond.

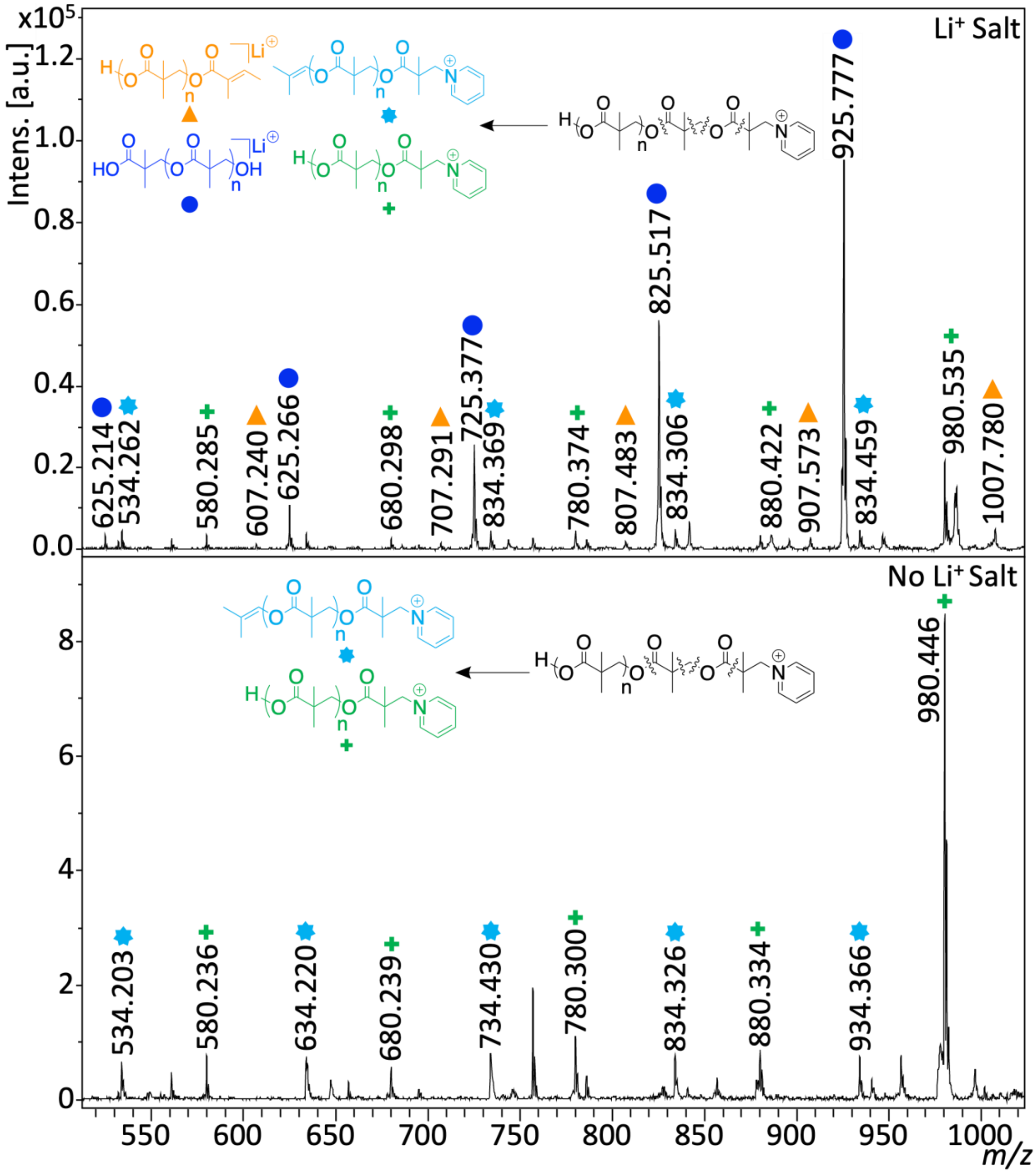

Figure SI 9. Tandem mass spectrometry of 10-mer poly(DMP)-pyr. The polymer chain fragments into various oligomers, which ionize either through the pyridinium ion or by forming adducts with Li^+ cations. The top spectrum shows the sample doped with Li^+ salt, while the bottom spectrum

is without Li⁺ doping. The orange alkene product can be produced as *E* or *Z* isomer, however, the one shown is more favorable.

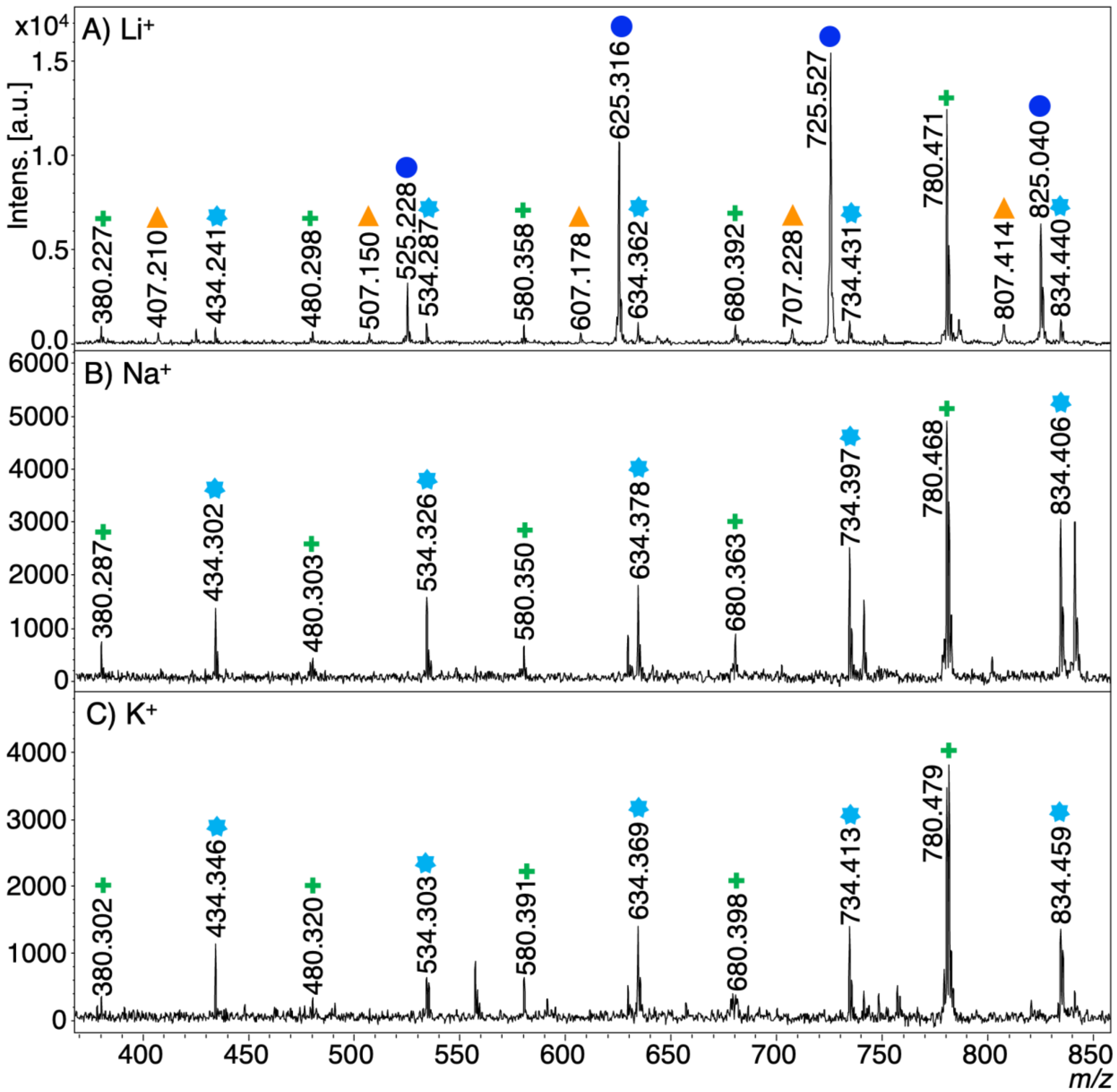

Figure SI 10. Tandem mass spectrometry of 8-mer poly(DMP)-pyr with A) Li^+, B) Na^+, and C) K^+ cation. The polymer chain fragments into various oligomers, which ionize either through the pyridinium ion or by forming adducts with Li^+ cation only. The additional fragments observed only in the Li^+ spectrum is attributed to precursor contamination from co-isolated poly(DMP)–pyr 8-

mer species bearing a COOLi chain end, enabled by the small mass difference of Li^+ and reduced isolation selectivity within the TIS window. These fragments (blue circles and orange triangles) are absent in the Na^+ and K^+ spectra, where larger mass differences allow cleaner precursor isolation.

Experimental Section

Materials

3-Chloro-2,2-dimethylpropanoic acid was obtained from Aaron Chemical. 2,2-Dimethyl-3-hydroxypropanoic acid (HPA) and 1,1,1,3,3,3-hexafluoro-2-propanol (HFIP), and magnesium sulfate were acquired from Oakwood Chemical. *Para*-toluenesulfonic acid, sodium methoxide, and lithium trifluoroacetate (LiTFA) were acquired from Sigma Aldrich. *Trans*-2-[3-(4-*tert*-Butylphenyl)-2-methyl-2-propenylidene]malononitrile (DCTB) was acquired from TCI Chemicals. Chloroform, methanol, tetrahydrofuran, and potassium carbonate were all acquired from Thermo Fisher Scientific. Deionized water was obtained using Philadelphia Scientific filter. All chemicals were utilized in their received condition. 3-Bromo-2,2-dimethylpropanoic acid and 3,3-dimethylpropiolactone were prepared in the lab.

Synthesis of 3-bromo-2,2-dimethylpropanoic acid

3-Bromo-2,2-dimethylpropanoic acid monomer was synthesized by the modified literature procedure.[1] 2,2-Dimethyl-3-hydroxypropionic acid (HPA) (35 g, 0.29 mol, 1 eq) and 350 mL hydrobromic acid was added to a 1000 mL round bottom-ed flask. The round bottom flask was heated in an oil bath at 130 ^oC for ~18 hours. The reaction mixture was then distilled at 160 ^oC. The product crystalizes in the receiving flask with water. The mixture was then extracted with

petroleum ether, organic phase was washed with deionized water once, dried with magnesium sulfate, and concentrated in vacuo to obtain white crystals (35.79 g, 66% yield).

Synthesis of 3,3-dimethylpropiolactone

3,3-Dimethylpropiolactone monomer was synthesized by the modified literature procedure.[2] 3-Bromo-2,2-dimethylpropanoic acid (Br-PA) (40 g, 0.22 mol, 1 eq) and 350 mL chloroform was added to a 500 mL round bottom-ed flask. The round bottom flask was submerged in a pre-heated oil bath at 50 ^oC. Once the monomer dissolved, 3M sodium hydroxide solution (8.90 g, 0.22 mol, 1 eq) was added to the flask. After 6 h, the reaction mixture was cooled to room temperature, extracted with chloroform (30 mL x 3), and the organic phase was washed with brine, dried with magnesium sulfate, and concentrated in vacuo (35 ^oC, 280 mbar). The resultant residue was purified by distillation (~10 mmHg, 50 ^oC, could be heated to 70-90 ^oC until the end) to obtain the monomer (12.0 g, 54.5% yield) as a colorless oil. The isolated monomer was stored in a under liquid nitrogen.

Synthesis of linear Poly(DMP)-OH

2,2-Dimethyl-3-hydroxypropionic acid (2.00 g, 16.9 mmol, 1 eq) was added to a 25 mL double-neck round bottom flask. The round bottom flask was submerged in a pre-heated oil bath at 140 ^oC. Once HPA melted completely *para*-toluenesulfonic acid (175 mg, 0.102 mmol, 0.06 eq) was added. To water from the product, the round bottom-ed flask was purged with N_2 gas and attached to drierite. The reaction was then allowed to react for 15 mins. The product was dissolved in 1,1,1,3,3,3-hexafluoro-2-propanol and precipitated in methanol. After filtered, the

white solid was converted to a powder using mortar and pestle and dried in vacuum desiccator overnight. Repeat unit = 100.05 Da, M_n= 850 Da, M_w= 950 Da, and Đ = 1.115 by MALDI-ToF MS.

Synthesis of cyclic Poly(DMP)

2,2-Dimethyl-3-hydroxypropionic acid (2.00 g, 16.9 mmol, 1 eq) was added to a 25 mL double-neck round bottom flask. The round bottom flask was submerged in a pre-heated oil bath at 230 ^oC. Once HPA melted completely *para*-toluenesulfonic acid (175 mg, 0.102 mmol, 0.06 eq) was added. To complete remove all the product water, the round bottom-ed flask was above the boiling point of water, was purged with N_2 gas and attached to drierite. The reaction was then allowed to react for 120 mins. The product was dissolved in 1,1,1,3,3,3-hexafluoro-2-propanol and precipitated in methanol. After filtered, the white solid was converted to powder using mortar and pestle and dried in vacuum desiccator overnight. Repeat unit= 100.05 Da, M_n= 1230 Da, M_w= 1350 Da, and Đ= 1.090 by MALDI-ToF MS.

Synthesis of linear Poly(DMP)-Cl

3-Chloro-2,2-dimethylpropanoic acid (100 mg, 0.740 mmol, 1 eq) and 3 mL dry dimethylformamide (DMF) was added to a 5 mL round bottom-ed flask. The round bottom flask was submerged in a pre-heated oil bath at 50 ^oC. Once the monomer dissolved, potassium carbonate (203 mg, 1.48 mmol, 2 eq) was added. The round bottom flask was purged with N_2 gas, and the reaction was then allowed to stir for 6 hours. The reaction was filtered to remove the solid base. DMF was removed by rotary evaporator. The product was then dissolved in chloroform, washed with deionized water, and dried with magnesium sulfate. It was then filtered,

rotary evaporated, and dried in vacuum desiccator overnight. Repeat unit = 100.05 Da, M_n= 1140 Da, M_w= 1250 Da, and Đ = 1.091 by MALDI-ToF MS.

Synthesis of linear Poly(DMP)-Br

3-Bromo-2,2-dimethylpropanoic acid (200 mg, 1.10 mmol, 1 eq) and 10-20 mL dry toluene was added to a 25 mL round bottom flask. The round bottom-ed flask was submerged in a pre-heated oil bath at 100 ^oC. Once the monomer dissolved, sodium methoxide (238 mg, 4.42 mmol, 4 eq) was added. The round bottom flask was purged with N_2 gas, and the reaction was then allowed to stir for 2 hours. The reaction was filtered to remove the solid base. The product was then dissolved in chloroform, washed with deionized water, and dried with magnesium sulfate. It was then filtered, rotary evaporated, and dried in vacuum desiccator overnight. Repeat unit = 100.05 Da, M_n= 1050 Da, M_w= 1100 Da, and Đ = 1.057 by MALDI-ToF MS.

Synthesis of linear Poly(DMP)-pyr

3,3-Dimethylpropiolactone (0.500 mL, 5.00 mmol, 1 eq) was syringed into a 25 mL clean and dry 2-neck round bottom flask. The system was kept under nitrogen. Then pyridine (40 µL, 0.050 mmol, 0.1 eq) was syringed into the round bottom flask and the reaction was left to stir until the product solidified. The product was dissolved in 1,1,1,3,3,3-hexafluoro-2-propanol and precipitated in methanol. The orange filtrate was collected and dried in vacuum desiccator overnight. Repeat unit = 100.05 Da, M_n= 2270 Da, M_w= 2820 Da, and Đ = 1.240 by MALDI-ToF MS.

References

(1) Greene, J. L. Jr.; Hagemeyer, H. J. Jr. Sulfur-Containing Pivalic Acid Derivatives. I. Sulfonyldipivalic Acid. *J. Am. Chem. Soc.* **1955**, *77* (11), 3016–3018. https://doi.org/10.1021/ja01616a029.

(2) Zhou, L.; Zhang, Z.; Shi, C.; Scoti, M.; Barange, D. K.; Gowda, R. R.; Chen, E. Y.-X. Chemically Circular, Mechanically Tough, and Melt-Processable Polyhydroxyalkanoates. *Science* **2023**, *380* (6640), 64–69. https://doi.org/10.1126/science.adg4520.
